# Studies on Chemical Characterization of Ginkgo Amillaria Oral Solution and Its Drug–Drug Interaction With Piceatannol 3′-*O*-*β*-D-Glucopyranoside for Injection

**DOI:** 10.3389/fphar.2022.932646

**Published:** 2022-07-19

**Authors:** Zhenyan Yu, Xiaohan Hu, Lin Zhou, Huliang Chen, Yanchao Xing, Chunyue Han, Hui Ding, Lifeng Han, Guixiang Pan, Zhifei Fu

**Affiliations:** ^1^ State Key Laboratory of Component-Based Chinese Medicine, Tianjin University of Traditional Chinese Medicine, Tianjin, China; ^2^ Second Affiliated Hospital of Tianjin University of Traditional Chinese Medicine, Tianjin, China

**Keywords:** Ginkgo Amillaria oral solution, piceatannol-3′-O-β-D-glucopyranoside for injection, LC-MS/MS, drug–drug interactions, pharmacokinetics

## Abstract

Ginkgo Amillaria oral solution (GAO) is commonly used for the treatment of cardiovascular and cerebrovascular diseases in China. Piceatannol-3′-*O*-*β*-D-glucopyranoside for injection (PGI) is mainly used for the prevention and treatment of ischemic cerebrovascular diseases. With the spread of cerebrovascular disease, the possibility of combining the two drugs has increased; however, there is no research on the drug–drug interaction (DDI) between these two medicines. In this paper, an ultrahigh-performance liquid chromatography/quadrupole–orbitrap mass spectrometry (UHPLC/Q-Orbitrap MS) method was established to characterize the chemical constituents of GAO first; 62 compounds were identified or tentatively identified based on their retention time (RT), MS, and MS/MS data. Nine main compounds were determined by ultrahigh-performance liquid chromatography/triple quadrupole mass spectrometry (UPLC-QQQ-MS). Furthermore, incubation with liver microsomes *in vitro* was fulfilled; the results showed that GAO had a significant inhibitory effect on UGT1A9 and UGT2B7 (*p* < 0.05), and PGI was mainly metabolized by UGT1A9. The identification results of *in vivo* metabolites of PGI showed that PGI mainly undergoes a phase II binding reaction mediated by UDP-glucuronosyltransferase (UGT) and sulfotransferase (SULT) *in vivo*. Therefore, pharmacokinetic studies were performed to investigate the DDI between GAO and PGI. The results showed that the AUC (*p* < 0.05) and T_1/2_ (*p* < 0.05) of PGI *in vivo* were significantly increased when administered together with GAO, whereas the CL was significantly decreased (*p* < 0.05). The exploration of *in vitro* and *in vivo* experiments showed that there was a DDI between GAO and PGI.

## 1 Introduction

Cerebrovascular disease is a common cause of morbidity and mortality worldwide, affecting millions of people each year (Fadini et al., 2011; Heidenreich et al., 2011; Costantino et al., 2016; Tourancheau et al., 2019). Ginkgo Amillaria oral solution (GAO) is a prescription composed of ginkgo biloba extract (GBE) and armillaria. The chemical constituents of GBE include flavonoids, ginkgolides, and phenolic acids, whereas armillaria mainly contains amino acids, nucleosides, polysaccharides, and sesquiterpenes (Huang et al., 2017; Eisvand et al., 2020; Li et al., 2020; Zhong et al., 2021). GBE inhibits troponin concentration better than armillaria, whereas armillaria is better than GBE in anti-platelet activation and inhibiting the expression of inflammatory factors. Experiments showed that the combination of the two is more effective than any single one and has a wide range of targets (Guo et al., 2017). GAO has been reported to possess the functions of promoting blood circulation and removing blood stasis and anti-atherosclerosis activity (Yang et al., 2016). It is widely used for the treatment of cardiovascular and cerebrovascular diseases in China (Tian et al., 2017). Piceatannol-3′-*O*-*β*-D-glucopyranoside (PG) is one of the active compounds of dried root or rhizome of *Rheum lhasaense* A. J. Liet P. K. Hsiao. Studies have shown that it has various pharmacological activities, including anti-inflammatory, anti-arteriosclerosis, anti-tussive, and anti-asthmatic (Woo et al., 2013). Studies have shown that PG is widely distributed in rats, has a fast elimination rate and linear kinetics, and is mainly excreted by urine (Chai, 2019; Xu, 2020). Piceatannol-3′-*O*-*β*-D-glucopyranoside for injection (PGI) is an innovative new drug that is currently in the phase I stage of clinical trial (clinical approval: 2018L02062). It is mainly used for the prevention and treatment of ischemic cerebrovascular diseases. Both GAO and PGI have similar indications that increased the probability of combination in clinic; however, no research has been reported on the data of drug–drug interaction (DDI) on the combination of the two.

As we all know, DDI includes pharmacokinetic drug–drug interactions (PDDI) and pharmacodynamic drug–drug interactions, in which the latter is easily observed. Therefore, PDDI is the main concern in clinic. PDDI refers to that one drug would affect the pharmacokinetic behaviors of another one, including the absorption, distribution, metabolism, and excretion. The blood concentration of the target compound would increase or decrease significantly, which would affect its efficacy or toxicity. Therefore, if there is PDDI between two drugs, which could generate adverse reactions or endanger the patient’s life (Łój et al., 2017). The mechanism of DDI may be due to regulating the activity of metabolic enzymes or transporters (Scheen, 2007; Palatini and De Martin, 2016). For example, pretreatment of GBE (100 mg kg^−1^) for 10 days would significantly reduce the area under the concentration–time curve (AUC) and maximum plasma concentration (*C*_max_) of propranolol, and the mechanism was GBE pretreatment-mediated induction of CYP1A2, which accelerates the conversion to the parent drug to *N*-desisopropylpropranolol (Zhao et al., 2006). In another clinical study of ten unrelated healthy male volunteers, a single dose of GBE orally did not affect the pharmacokinetics of talinolol. However, repeated ingestion of GBE increased the *C*_max_ of talinolol by 36% and AUC_0-24_ by 26%. The reason was likely due to P-glycoprotein (P-gp)-mediated DDI (Fan et al., 2009).

In this research, an ultrahigh-performance liquid chromatography/quadrupole–orbitrap mass spectrometry (UHPLC/Q-Orbitrap MS) method was established to characterize the chemical constituents of GAO firstly. Furthermore, nine main compounds (rutin, astragalin, *p*-hydroxybenzoic acid, 3,4-dihydroxybenzoic acid, uridine, guanosine, ginkgolide A, ginkgolide C, and ginkgolide J) from GAO were determined by ultrahigh-performance liquid chromatography/triple quadrupole mass spectrometry (UPLC-QQQ-MS). In addition, *in vitro* liver microsome incubation and *in vivo* metabolite identification experiments of PG were carried out to investigate possible DDI between GAO and PG. At last, a pharmacokinetic model was constructed to investigate the PDDI of GAO and PG *in vivo*. Plasma concentrations of PG from 0 to 6 h were compared before and after 14 days of repeated administration of GAO. The results showed that the pharmacokinetic parameters of PG *in vivo* were significantly changed when administered together with GAO.

## 2 Materials and Methods

### 2.1 Chemicals and Reagents

Forty four reference substances, including five organic acids (**1**: gallic acid; **2**: 3,4-dihydroxybenzoic acid; **3**: *p*-hydroxybenzoic acid; **4**: D-quinic acid; and **5**: trans-aconitic acid); four ginkgolides (**6**: ginkgolide A; **7**: ginkgolide B; **8**: ginkgolide C; and **9**: ginkgolide J); five amino acids (**10**: L-arginine; **11**: valine; **12**: L-leucine; **13**: L-tyrosine; and **14**: L-aspartic acid); four nucleosides (**15**: cytidine; **16**: adenosine; **17**: guanosine; and **18**: uridine); two purines and two pyrimidines (**19**: adenine; **20**: guanine; **21**: uracil; and **22**: cytosine); 11 flavonoids (**23**: luteolin; **24**: naringenin; **25**: epicatechin; **26**: apigenin; **27**: rutin; **28**: catechin; **29**: astragalin; **30**: kaempferol; **31**: isorhamnetin; **32**: quercetin; and **33**: linarin); one compound from other class (**34**: 5-hydroxymethyl-2-furaldehyde); two stilbenes (**35**: PG and **36**: polydatin); specific substrates of UGT1A1, UGT1A3, UGT2B7, UGT1A19, and an internal standard (**37**: estradiol; **38**: chenodeoxycholic acid; **39**: zidovudine; **40**: 2,6-diisopropylphenol; and **41**: chlorpropamide); and three inhibitors of UGT1A1, UGT1A9, and UGT2B7 (**42**: pazopanib; **43**: niflumic acid; and **44**: fluconazole), were purchased from Shanghai Yuanye Biotechnology Co., Ltd. (Shanghai, China), National Institute for the Control of Pharmaceutical and Biological Products (Shanghai, China), Shanghai Shidander Biotechnology Co., Ltd. (Shanghai, China), and Chengdu Manster Biotechnology Co., Ltd. (Chengdu, China). Piceatannol-3′-*O*-*β*-D-glucopyranoside for injection (batch number: 20200530) was provided by Kunming Pharmaceutical Group Co., Ltd. (Yunnan, China), and GAO solution (batch number: 201149) was provided by Qionglai Tianyin Pharmaceutical Co., Ltd. (Sichuan, China). Rat liver microsomes (batch number: 0112041) were purchased from Huizhi Taikang Biotechnology Co., Ltd. (Beijing, China).

HPLC-grade acetonitrile and methanol were purchased from Fisher Scientific (Fair Lawn, NJ, United States). Formic acid and acetic acid were obtained from ACS (Wilmington, DE, United States) and Sigma-Aldrich (St. Louis, MO, United States), respectively. Deionized water was purified using a Milli-Q system (Millipore, Bedford, MA, United States).

### 2.2 Characterization of Chemical Constituents of Ginkgo Amillaria Oral Solution

#### 2.2.1 Sample Preparation

A total of 20 μl of GAO was precisely measured and transferred into a 1.5 ml centrifuge tube, and 980 μl of ultrapure water was added. The mixture was vortexed for 3 min and centrifuged at 13,200 × g under 4℃ for 10 min. For further LC-MS analysis, 3°μl of the supernatant was injected.

#### 2.2.2 Detection and Analysis Based on UHPLC/Q-Orbitrap MS

An Ultimate 3000 ultrahigh-performance liquid system (Thermo Fisher Scientific, San Jose, CA, United States) was used for separation. A Waters ACQUITY UPLC HSS T_3_ column (2.1 × 100 mm, 1.8 μm) was employed at 35°C with a flow rate of 0.3 ml min^−1^. The mobile phase consisted of water containing 0.1% FA (A) and acetonitrile (D). The following linear gradient was used: 0–2 min: 1–2.6% D; 2–3.5 min: 2.6–6% D; 3.5–3.8 min: 6–7.4% D; 3.8–5 min: 7.4–11% D; 5–6.5 min: 11–12.4% D; 6.5–8.6 min: 12.4–16.5% D; 8.6–16.6 min: 16.5–28.4% D; 16.6–20 min: 28.4–34% D; 20–23 min: 34–46% D; and 23–24 min: 46–60% D.

Data-dependent acquisition (DDA) was performed using the Q Exactive™ hybrid Q-Oritrap MS (Thermo Fisher Scientific, San Jose, CA, United States) in both negative and positive ion modes. The heated electrospray ionization (HESI) source parameters were set as follows: spray voltage (SV), 3.0 kV for negative mode, and 3.5 kV for positive mode; sheath gas pressure, 35 psi; aux gas pressure, 8 arb; capillary temperature (CT), 350℃; and aux gas heater temperature (AT), 400℃. A full MS/dd-MS^2^ (Top 5) scan method was applied for DDA mode. The scan range of full MS was *m/z* 100–1,500. The resolution of MS^1^ and MS^2^ was set as 70,000 and 17,500, respectively. The normalized collision energy (NCE) was set as 20/40/60 V, with an isolation width of 4.0 Da. The dynamic background exclusion time was 10 s, and the fragmentation information of more targeted co-eluting precursors could be recorded by setting the function of dynamic exclusion. In addition, the function of “*If idle-pick others*” was chosen to record the MS^2^ spectra of several nontarget compounds at the same time.

### 2.3 Characterization of Nine Chemical Constituents in Ginkgo Amillaria Oral Solution

#### 2.3.1 Standard Solutions

Standard stock solutions of ginkgolide A; ginkgolide C; ginkgolide J; *p*-hydroxybenzoic acid; 3,4-dihydroxybenzoic acid; uridine; guanosine; and linarin (IS) were prepared at a concentration of 1 mg mL^−1^ in 50% methanol-H_2_O (*v/v*). Rutin and astragalin were prepared at concentrations of 0.5 mg mL^−1^ and 1 mg mL^−1^ in 50% methanol-H_2_O (*v/v*), with heated and sonicated until completely dissolved. All the solutions were stored at 4℃ for further analysis.

#### 2.3.2 Detection and Analysis Based on UPLC-QQQ-MS

An ultrahigh-performance liquid chromatography (Waters, United States) was performed for separation. A Waters ACQUITY UPLC HSS T_3_ column (2.1 × 100 mm, 1.8 μm) was employed at 35℃ with a flow rate of 0.3 ml min^−1^. The mobile phase consisted of acetonitrile (A) and water containing 0.1% FA (B). The following linear gradient was used: 0–1 min: 1–2.6% A; 1–3 min: 2.6–6% A; 3–4 min: 6–11% A; 4–5 min: 11–12.4% A; 5–6 min: 12.4–14% A; 6–6.5 min: 14–21% A; 6.5–8.5 min: 21–24% A; 8.5–10 min: 24–30% A; 10–12 min: 30–36% A; 12–12.1 min: 36–1% A; and 12.1–14 min: 1% A.

Multiple reaction monitoring (MRM) was performed using the QQQ-MS model Xevo TQ-S (Waters, United States). Electrospray ionization (ESI) was set as follows: capillary voltage, 3.0 kV; desolvation temperature: 650℃; and desolvation gas flow: 900 L/h (N_2_, purity 99.9%); data acquisition and data analysis were completed using Masslynx (4.1), and the ion pairs and mass spectrometry parameters of the nine compounds are shown in Table 1. Linarin was chosen as the internal standard (IS) since it is a flavonoid compound and does not exist in GAO.

**TABLE 1:** Transition and mass spectrometry parameters of nine compounds.

Comp	RT (min)	Detect mode	Precursor ions (*m/z*)	Product ions (*m/z*)	Cone voltage (V)	Collision energy (V)
Uridine	2.62	ESI+	245.10	113.07	16	18
Guanosine	3.99	ESI+	284.00	152.03	10	8
3,4-Dihydroxybenzoic acid	5.73	ESI−	153.06	108.96	40	20
*p*-hydroxybenzoic acid	7.06	ESI−	137.00	93.03	46	16
Rutin	9.16	ESI−	609.12	271.09	78	52
Ginkgolide J	9.90	ESI−	423.17	73.05	64	30
Ginkgolide C	10.04	ESI−	439.29	143.02	2	30
Astragalin	10.23	ESI−	447.07	284.13	66	40
Linarin(IS)	12.17	ESI−	591.27	283.14	62	24
Ginkgolide A	12.46	ESI−	407.17	319.21	50	12

#### 2.3.3 Method Validation

The method was validated in terms of linearity, precision, stability, recovery, and repeatability. Linearity was required to be greater than 0.999 (*r* ^2^); the RSDs for precision, stability, recovery, and repeatability were less than 5%.

### 2.4 Inhibitory Effect of GAO on UDP-Glucuronosyltransferase

#### 2.4.1 Microsomal Incubations

The incubation mixtures consisted of 1 mg mL^−1^ of rat liver microsomes; 1 mmol L^−1^ of UDPGA; 100 μg mL^−1^, 10 μg mL^−1^, and 1 μg mL^−1^ of GAO; tris-HCl (pH = 7.4) and specific substrates (estradiol (10 μmol L^−1^)); chenodeoxycholic acid (10 μmol L^−1^); 2,6-diisopropylphenol (100 μmol L^−1^); and zidovudine (10 μmol L^−1^). The total volume was 100 μl. After pre-incubation at 37℃ for 5° min, reactions were initiated by the addition of 1 mmol L^−1^ UDPGA and were incubated for 1 h at 37℃ in a water bath. The reactions were terminated by the addition of 100 μl ice acetonitrile containing chlorpropamide (400 ng mL^−1^, IS) and centrifuged at 13,200 × g under 4℃ for 10 min. An aliquot of the supernatant was injected into LC-MS/MS for the determination of substrates.

#### 2.4.2 Detection and Analysis Based on UPLC-QQQ-MS

An ultrahigh-performance liquid chromatography (Waters, United States) was used for separation and detection. A Waters ACQUITY UPLC HSS T_3_ column (2.1 × 100 mm, 1.8 μm) was employed at 35℃ with a flow rate of 0.3 ml min^−1^. The mobile phase consisted of acetonitrile (A) and water containing 0.1% FA (B). The injection volume of each sample was 3 μl. The following linear gradient was used: 0–4.5 min: 30–90% A and 4.5–5.5 min: 95% A. MS conditions were the same as those in “2.3.2.” The MRM transitions, cone voltage, and collision voltage of the target compounds and IS are shown in Supplementary Table S1.

### 2.5 Metabolism of PG Based on UGTs

#### 2.5.1 Microsomal Incubations

The incubation mixtures consisted of 1 mg mL^−1^ of rat liver microsomes, 1 mmol L^−1^ of UDPGA and 10 μmol L^−1^ of PG, tris-HCl (pH = 7.4) and inhibitor (pazopanib (50 μmol L^−1^)), niflumic acid (10 μmol L^−1^), and fluconazole (50 μmol L^−1^). The incubation system was the same as that in “2.4.1.,” whereas polydatin (20 μg mL^−1^) was selected as the IS. Detection and analysis conditions based on UPLC-QQQ-MS were the same as those in “2.4.2.” The MRM transitions, cone voltage, and collision voltage of the target compounds and IS are shown in Supplementary Table S1.

### 2.6 Metabolites of PG *In Vivo*

#### 2.6.1 Animals

Nine Sprague–Dawley (SD) male rats (200 ± 20 g) were purchased from Beijing Vital River Laboratory Animal Technology Co., Ltd. (license number: SCXK (Beijing) 2016-0006).

#### 2.6.2 Collection of Experimental Samples

The plasma samples were the same as in the pharmacokinetic experiment; PGI (9 mg kg^−1^) was injected into the tail vein of three rats to collect the bile samples for 0–6 h, and saline (9 mg kg^−1^) was injected into the tail vein of three rats to collect the normal bile samples. PGI (9 mg kg^−1^) was injected into the tail vein of three rats to collect urine (0–4 h) samples and feces (0–6 h) samples after collecting the blank urine and feces samples. All samples were stored at −80℃ for further analysis.

#### 2.6.3 Sample Extraction Procedure

Plasma samples: 40 μl of plasma samples at different time points were taken and mixed together in a 2 ml centrifuge tube; 1,280 μl of ice acetonitrile was added. The mixture was vortexed for 3 min and centrifuged at 13,200 × g under 4℃ for 10 min. The supernatant was transferred to another centrifuge tube and dried with nitrogen, re-dissolved with 60 μl of 80% methanol-H_2_O (*v/v*). For further LC-MS analysis, 3 μl of the supernatant was injected.

Bile samples: 100 μl of bile sample was taken using a 0.22 μm filter, the mixture was vortexed for 3 min, and centrifuged at 13200 × g under 4℃ for 10 min. For further LC-MS analysis, 3 μl of the supernatant was injected.

Fecal samples: 100 mg of the sample was weighed into a 1.5 ml centrifuge tube, 400 μl of ice acetonitrile was added, and the mixture was vortexed for 3 min and centrifuged at 13,200 × g under 4℃ for 10 min. For further LC-MS analysis, 3 μl of the supernatant was injected.

Urine sample: 600 μl of urine sample was taken out, and 2,400 μl of ice acetonitrile was added. The mixture was vortexed for 3 min and centrifuged at 13,200 × g under 4℃ for 10 min. The supernatant was transferred to another centrifuge tube and dried with nitrogen, re-dissolved with 60 μl of 80% methanol-H_2_O (*v/v*). For further LC-MS analysis, 3 μl of the supernatant was injected.

#### 2.6.4 Detection and Analysis Based on UHPLC/Q-Orbitrap MS

An ultrahigh-performance liquid chromatography-mass spectrometer (Waters, United States) was performed for separation and detection. A Waters ACQUITY UPLC HSS T_3_ column (2.1 × 100 mm, 1.8 μm) was employed at 30℃ with a flow rate of 0.3 ml min^−1^. The mobile phase consisted of acetonitrile (B) and water containing 0.1% FA (D). The following linear gradient was used: 0–0.5 min: 5–7% B; 0.5–2.5 min: 7–8.8% B; 2.5–5.5 min: 8.8–16.9% B; 5.5–14 min: 16.9–24.7% B; 14–23 min: 24.7–52% B; and 23–30 min: 52–95% B.

DDA was performed using the Q Exactive™ hybrid quadrupole–Orbitrap mass spectrometer both in negative and positive mode. HESI source parameters were set as follows: spray voltage, 3.0 kV for negative mode, 3.5 kV for positive mode; sheath gas pressure, 35 psi; aux gas pressure, 8; capillary temperature, 400℃; and aux gas heater temperature, 450℃. A full MS/dd-MS^2^ (Top 5) scan method was applied for DDA. The scan range of full MS was *m/z* 100–1,500. The resolution of MS^1^ and MS^2^ was set as 70,000 and 17,500, respectively. NCE was set as 30/40/50 V, with an isolation width of 4.0 Da.

### 2.7 Effects of GAO on the Pharmacokinetics of PGI

#### 2.7.1 Sample Extraction Procedure

A total of 100 μl of rat plasma sample and 10 μl of polydatin solution (IS) were mixed with 400 μl of ice acetonitrile; the mixture was vortexed for 3 min and centrifuged at 13,200 × g under 4℃ for 20 min. The supernatant was transferred to another centrifuge tube and dry with nitrogen, re-dissolved with 100 μl of 80% methanol-H_2_O (*v/v*). The mixture was vortexed for 3 min and centrifuged at 13,200 × g under 4℃ for 20 min. For further LC-MS analysis, 3 μl of the supernatant was injected.

#### 2.7.2 Detection and Analysis Based on UPLC-QQQ-MS

An ultrahigh-performance liquid chromatography-mass spectrometer (Waters, United States) was used for separation and detection. A Waters ACQUITY UPLC HSS T_3_ column (2.1 × 100 mm, 1.8 μm) was employed at 35℃ with a flow rate of 0.3 ml min^−1^. The mobile phase consisted of acetonitrile (A) and water containing 0.1% FA (B). The following linear gradient was used: 0–4 min: 25–95% A; 4–4.5 min: 95–25% A; and 4.5–6 min: 25% A.

MRM was performed using QQQ-MS model Xevo TQ-S (Waters, United States). ESI was set as follows: capillary voltage, 3.0 kV; desolvation temperature, 450℃; and desolvation gas flow, 900 L/h (N_2_, purity 99.9%). The MRM transitions (precursor → product) of PG and polydatin (IS) were *m/z* 405.16 → *m/z* 243.18 and *m/z* 389.16 → *m/z* 227.20, respectively. The cone voltage of PG and polydatin were 80 and 84 V, respectively. The collision energy of PG and polydatin was 30 and 20 V, respectively.

#### 2.7.3 Method Validation

UPLC-MS/MS method validation was performed according to the FDA bioanalytical method validation guidance. The method was validated in terms of linearity, accuracy, precision, extraction recovery, matrix effect, and stability.

#### 2.7.4 Animals

Sixteen SD male rats (200 ± 20 g) were purchased from Beijing Vital River Laboratory Animal Technology Co., Ltd. (license number: SCXK (Beijing) 2016-0006). All rats were reared adaptively in a suitable environment for 1 week with freely available food and water. The temperature was maintained at 22℃, and the light was maintained for 12 h in a day.

#### 2.7.5 Pharmacokinetic Study

The rats were randomly divided into two groups, viz., the control group (*n* = 8) and the combined group (*n* = 8). For the control group, 9 mg kg^−1^ of PGI was injected into the tail vein of rats, whereas the same dosage of PGI was injected after 14 days of repeated GAO ingestion (51 mg kg^−1^) for the combined group. The dosage administered to rats is in accordance with clinical dosage. After administration, blood samples were obtained from each rat at 0.03, 0.08, 0.16, 0.33, 0.5, 0.75, 1, 2, 3, 4, and 6 h. The blood sample was centrifuged at 6,600 × g under 4℃ for 10 min, and the supernatant was stored at −80℃ for further analysis.

## 3 Results

### 3.1 Chemical Constituents in GAO

**FIGURE 1:**
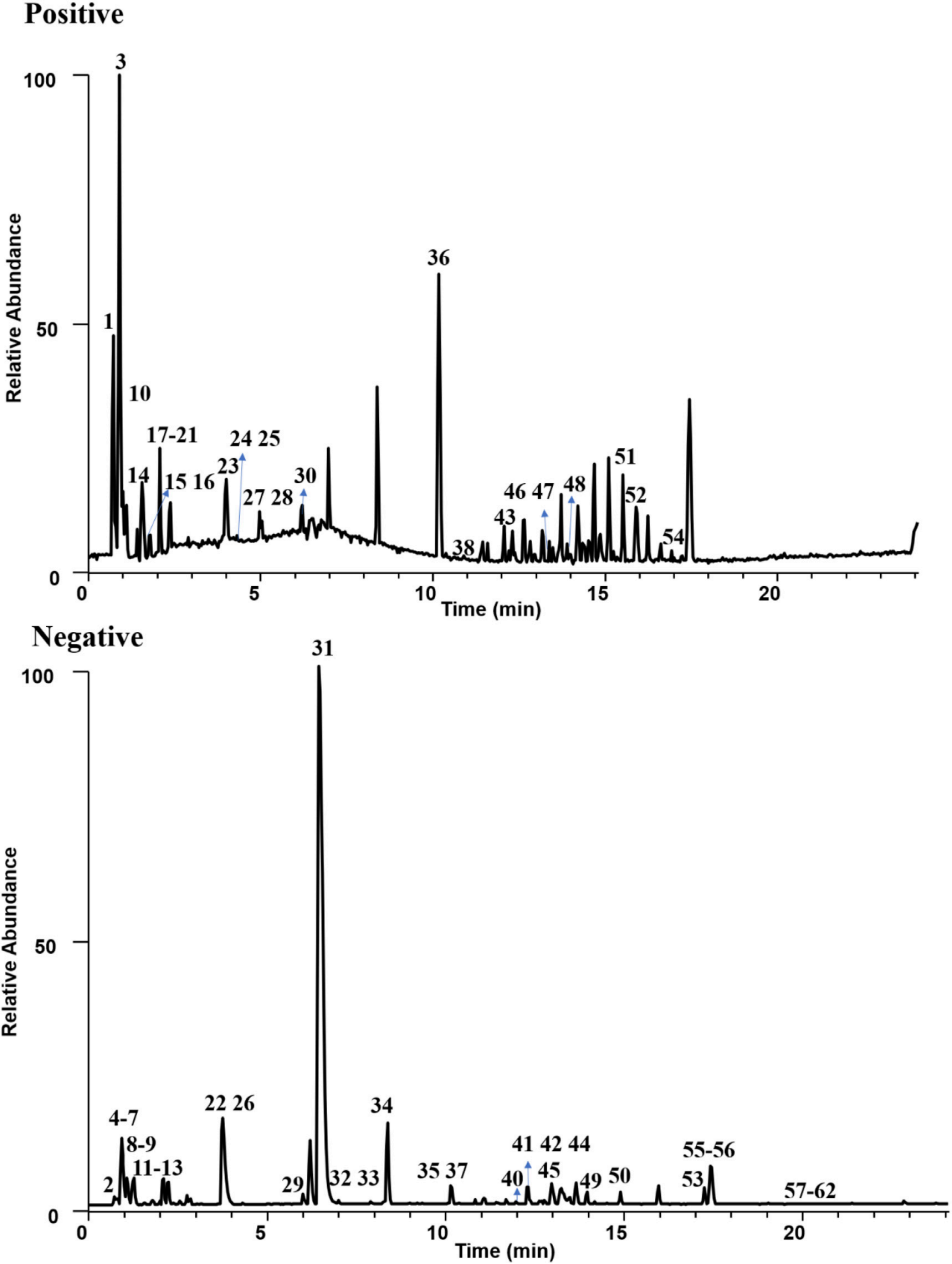
Total ion chromatography of GAO sample.

The total ion current diagram of GAO in positive and negative ion modes is shown in Figure 1. All the raw data acquisition was performed using Xcalibur 4.0 (Thermo Fisher Scientific, United States). The quasi-molecular ions were controlled within ± 5 ppm according to the elemental composition provided by the software.

A total of 62 chemical constituents were identified; 26 compounds were identified in positive ion mode, and 36 compounds were identified in negative ion mode, including 10 amino acids, 19 flavonoids, 2 phenolic acids, 4 nucleosides, 2 purines, 2 pyrimidines, 4 ginkgolides, and 19 other compounds. Among them, 34 compounds were compared with standards (including RT, quasi-molecular ion mass, and fragment ion information), and 28 compounds were identified based on databases and related literature. The details are shown in Supplementary Table S2. We took **Comp. 40** (Supplementary Table S2) as examples to demonstrate analysis of the chemical composition. The molecular ions of [M-H]^−^ at *m/z* 609.14539 ([C_27_H_30_O_16_]^−^) were observed in the MS^1^ spectrum. The fragment ions of *m/z* 301.03131, 151.00230, 273.03528, 229.04823, 211.03793, 255.02905, 227.03397, and 211.03793 were observed. The compound was tentatively identified as rutin. The MS/MS spectra and possible fragmentation pathways are exhibited in Supplementary Figure S1.

In this experiment, an efficient UHPLC/Q-Orbitrap MS method was established to clarify the diverse chemical constituents of GAO. First, the liquid phase conditions were optimized, which includes the optimization of the chromatographic column, mobile phase, and column temperature. The HSS T_3_ column was selected according to the number of peaks and the resolution (Supplementary Figure S2). Peak shape could be improved when different proportions of acid are added to the water phase (Supplementary Figure S3); therefore, the mobile phase consisted of acetonitrile and water containing 0.1% FA. Different column temperature including 30℃, 35℃, and 40℃, was optimized, respectively. It was found that the best resolution could be obtained at 35℃ (Supplementary Figure S4).

The main bioactive compounds of GAO, representing different structure subclasses, were selected as qualitative indicators, namely, rutin, isoquercitrin, kaempferol-3-*O*-rutinoside, isorhamnetin-3-*O*-rutinoside (flavonoids) and ginkgolides A, B, C (ginkgolides), and 3,4-dihydroxybenzoic acid (phenolic acids). According to the corresponding peak areas of the eight types of components under different source parameter settings, the optimal mass spectrometry parameters were determined. The SV (2.5, 3.0, and 3.5 kV), the CT (200℃, 250℃, 300℃, and 350℃), and the AT (250℃, 300℃, 350℃, and 400℃) were optimized, respectively. The results are shown in Supplementary Figure S5.

Six types of components of GAO (rutin, kaempferol-3-*O*-rutinoside, ginkgolides A, ginkgolides B, 3,4-dihydroxybenzoic acid, and adenosine) were used to observe MS^2^ fragmentation behaviors to identify which were best to show clearly the diversity of products ions. NCE (10/20/30 V, 10/20/40 V, 20/30/40 V, and 20/40/60 V) were optimized, and the results are shown in Supplementary Figure S6. The blank solution control is shown in Supplementary Figure S7. The final mass spectrometry conditions SV, CT, AT, and NCE were determined as 3.5 kV, 350℃, 400℃, and 20/40/60 V, respectively.

### 3.2 Characterization of Nine Chemical Constituents in GAO

#### 3.2.1 Method Validation

The results showed that *r* ^2^ was all greater than 0.999 for linearity, and LODs and LOQs ranged from 0.1 ng ml^−1^ to 8 ng ml^−1^ and 1.4–16 ng ml^−1^, respectively (Supplementary Table S3). There was a good resolution and no impurity interference in the MRM spectrum (Supplementary Figure S8). The RSD of intraday and interday precision for each compound was less than 4.88%, and the RSDs of stability and repeatability were within 3.59% and 3.81%, respectively (Supplementary Table S4). The sample recoveries of the nine components were between 94.78 and 104.96%, and the RSDs were between 0.30% and 4.26% (Supplementary Table S5).

#### 3.2.2 Determination of Sample Content

**FIGURE 2:**
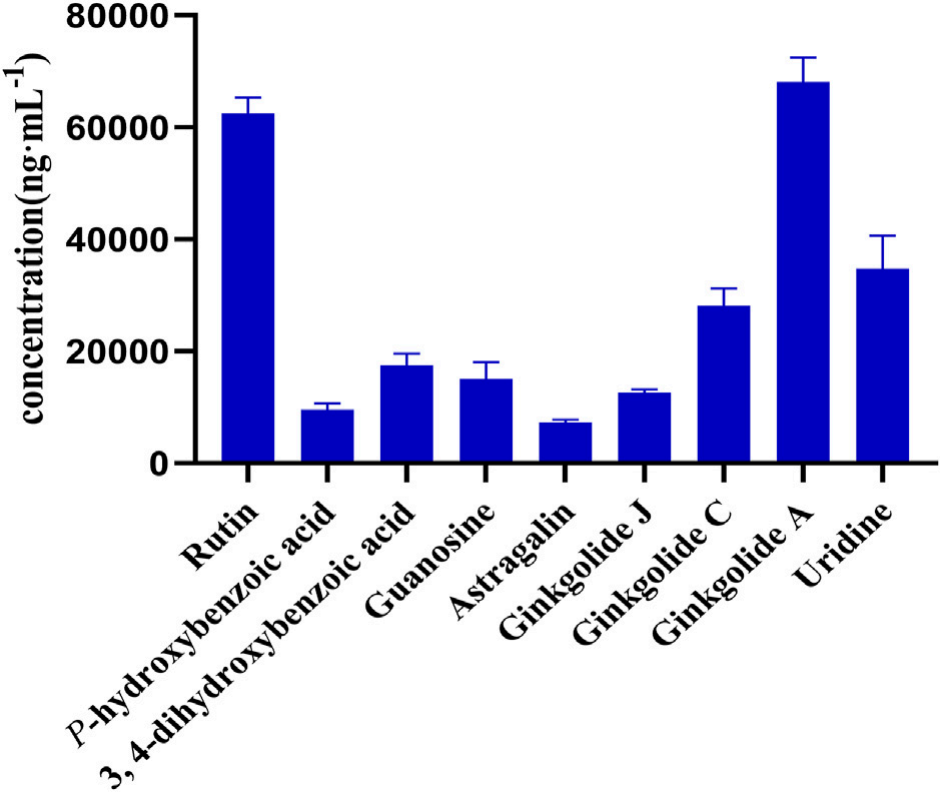
Content of nine compounds in 13 batches of GAO.

The test solution (*n* = 3) was prepared according to the method under “2.2.1.” and was measured according to the analytical method under “3.1.” The content measurement results are shown in Figure 2. More detailed information is provided in Supplementary Figure S9 and Supplementary Table S6.

A UPLC-MS/MS method was established and applied to 13 batches of GAO; the nine compounds were rutin, astragalin, *p*-hydroxybenzoic acid, 3,4-dihydroxybenzoic acid, uridine, guanosine, ginkgolide A, ginkgolide C, and ginkgolide J. The result showed that the contents of flavonoids, ginkgolides, and adenosine were higher, and the contents of uridine and guanosine were quite unstable in different batches.

### 3.3 Liver Microsome Incubation *In Vitro*

#### 3.3.1 Method Validation

The linear of five compounds were obtained, with all *r* ^2^ greater than 0.99. The LODs and LOQs ranged from 0.1 ng ml^−1^ to 10 ng ml^−1^ and 1–20 ng ml^−1^, respectively (Supplementary Table S7). The RSD of repeatability and precision were within 4.90% and 3.55% (Supplementary Table S8). The MRM spectra of eight compounds are shown in Supplementary Figures S10, S11.

#### 3.3.2 Liver Microsomes Incubation *In Vitro*

**FIGURE 3:**
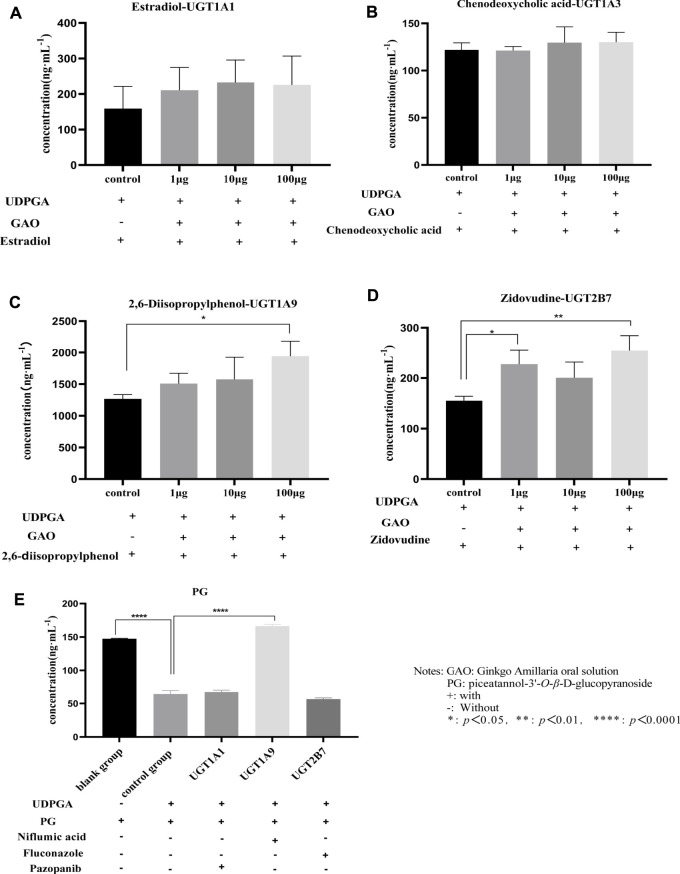
Incubation of liver microparticles with GAO and PG **(A)**: estradiol; **(B)**: chenodeoxycholic acid; **(C)**: 2,6-diisopropylphenol; **(D)**: zidovudine; and **(E)**: PG).

The results of incubation of liver microsomes *in vitro* are shown in Figures 3A–D. GAO has a significant inhibitory effect on UGT1A9 and UGT2B7 (*p* < 0.05). Further studies showed that PG was mainly metabolized by UGT1A9 (*p* < 0.0001), and UGT1A1 and UGT2B7 contributed little or no participation (*p* > 0.05), as shown in Figure 3E. The results of experiments *in vitro* indicated that DDI might occur when GAO and PGI were used in combination.

### 3.4 Metabolites of PG *In Vivo*

#### 3.4.1 Identification of Metabolites in Plasma

**FIGURE 4:**
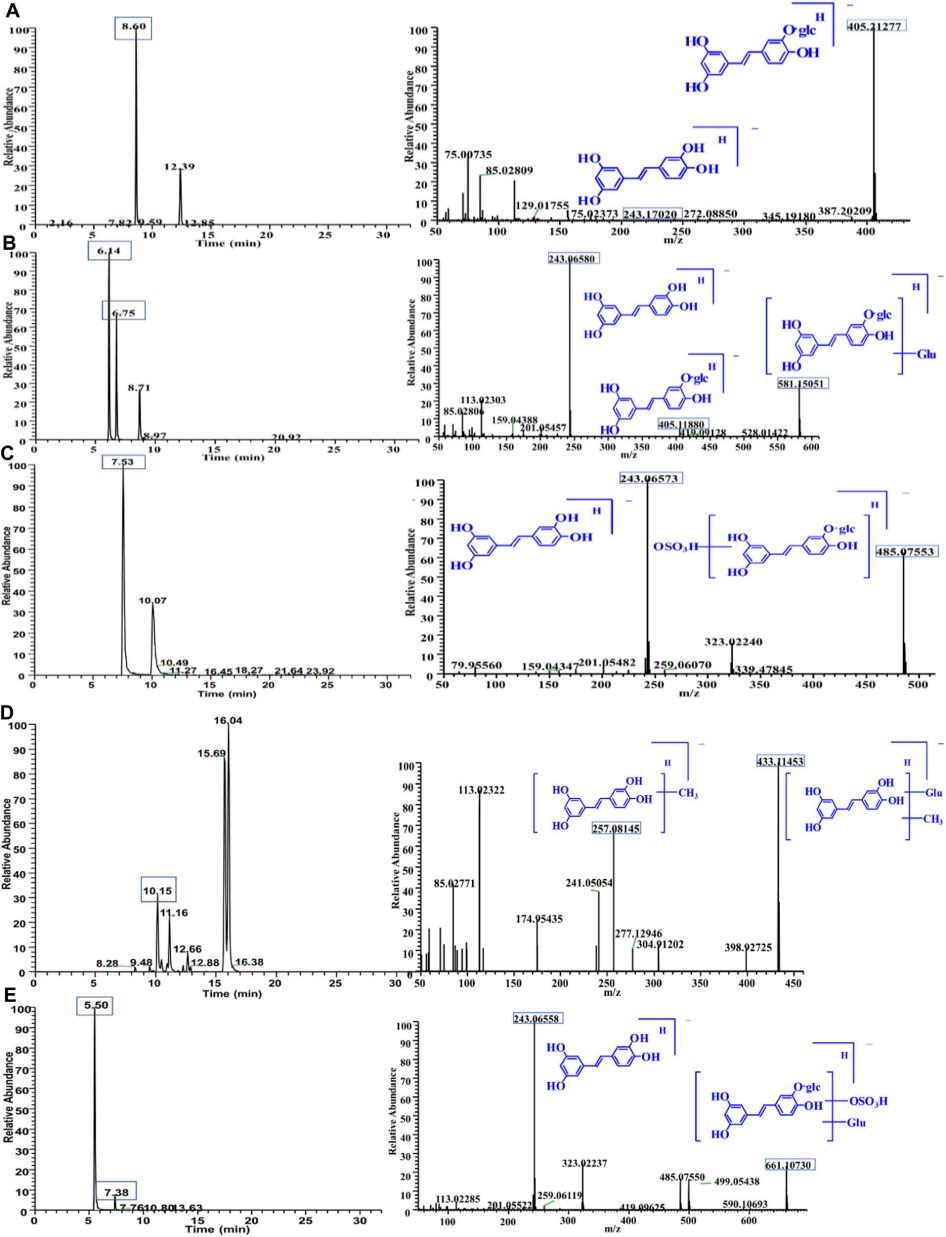
Chromatogram and MS/MS spectra of metabolites of PG *in vivo* **(A)**: M0 = 405.11838; **(B)** M1 = 581.15051; **(C)** M2 = 485.07553). Chromatogram and MS/MS spectra of metabolites of PG *in vivo* **(D)**: M3 = 433.11453; **(E)**: M4 = 661.10730).

PG was detected in plasma, which was named M0. The mass spectrum is shown in Figure 4A. The precursor ions existed in the form of [M-H]^-^ (*m/z* 405.21277, retention time (RT) = 8.60 min). The fragment ions at *m/z* 243.17020 corresponded to the loss of one glucose residue from PG, which was piceatannol. The *m/z* 581.15051 and 581.15033 were observed in the MS^1^ spectrum, and their RTs were 6.14 and 6.75 min, which were named M1-1 and M1-2, respectively. It is speculated that its molecular formula is C_26_H_30_O_15_; the fragments at *m/z* 581.15051 and 581.15033 corresponded to PG bound to a glucuronide; the fragment ions of *m/z* 405.11880 and 243.06580 were PG and piceatannol, respectively. The results indicated that these metabolites were glucuronide conjugates, and the mass spectrum is shown in Figure 4B. Log P (fat–water partition coefficient) and CLog P (theoretical value of fat–water partition coefficient) were calculated using Chem Draw 14.0 (Cambridge Soft Corp.). The lower the value, the stronger the hydrophilicity. Therefore, we determined that RT = 6.14 min was M1-1 and RT = 6.75 min was M1-2. The *m/z* 485.07553 and 433.11453 were observed from the chromatogram, and fragment ions are shown in Figures 4C,D. A total of four metabolites were identified in plasma, and the detected metabolites were all phase II metabolites, as shown in Supplementary Table S9.

#### 3.4.2 Identification of Metabolites in Bile

The *m/z* 661.10730 and 661.10724 were observed from the chromatogram; the RTs were 5.75 and 7.39 min, which were named M4-1 and M4-2, respectively. These molecular ions corresponded to increase one C_6_H_8_O_9_S residue on PG. The fragment ions of 243.06558 were observed. It is speculated that its molecular formula was C_26_H_30_O_18_S, indicating that these metabolites were sulfate–glucuronide conjugates, and the mass spectrum is shown in Figure 4E. A total of five metabolites were identified in bile, all of which were reaction products combined with phase II, as shown in Supplementary Table S10.

**FIGURE 5:**
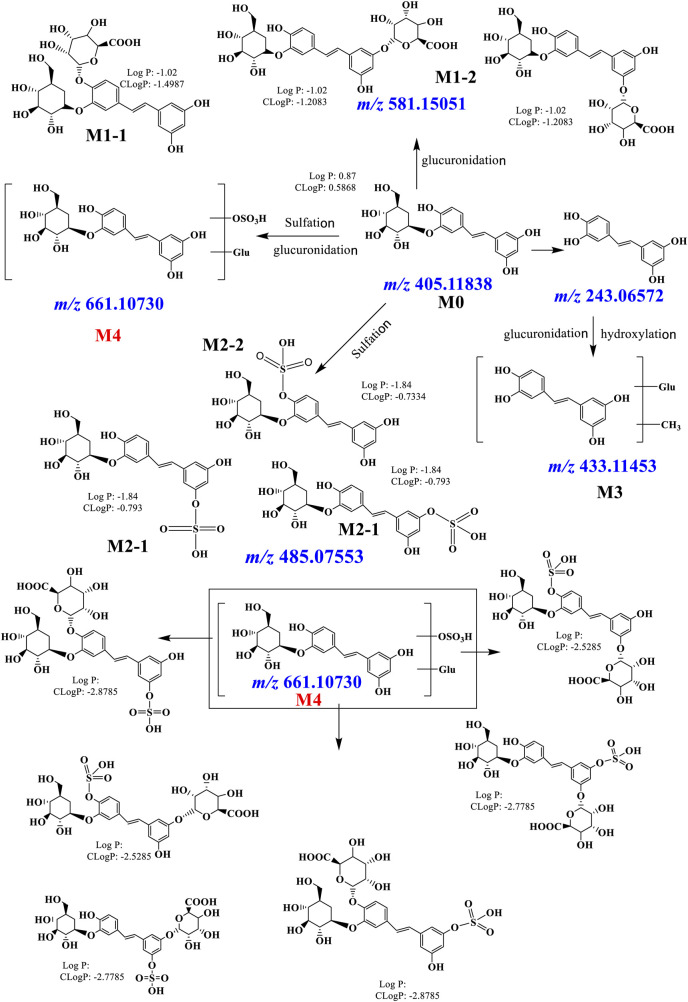
Possible metabolic pathways of PG *in vivo*.

Studies showed that 40.0 mg kg^−1^ of PG was administered to rats through tail vein and intragastric administration. No metabolites were detected in fecal samples. The prototype of PG in rats is mainly excreted through urine, and its main excretion form should be metabolites (Xu, 2020). The identification results of *in vivo* metabolites of PG showed that PG mainly undergoes a phase II binding reaction mediated by UGT and SULT *in vivo*. The pathways mainly include glucuronide conjugates, sulfate conjugates, sulfate–glucuronide conjugates, and glucuronic after piceatannol methylation. The metabolic pathways are shown in Figure 5 **.**

UGTs are involved in the metabolism and detoxification of drugs and the balance of the internal environment, which can catalyze the metabolic clearance of some endogenous substances and various exogenous substances (Tourancheau et al., 2018). Literature reported that the chemical components of GAO regulate CYP enzymes and UGT enzymes (Jiang and Hu, 2012; Cao et al., 2017). The results of *in vitro* liver microsomes incubation also proved that GAO has a significant inhibitory effect on UGT1A9 and UGT2B7.

### 3.5 Effects of GAO on the Pharmacokinetics of PGI

#### 3.5.1 Method Validation

The result of specificity is shown in Supplementary Figure S12. The RTs of PG and polydatin were 1.47 and 1.70 min, respectively. Their chromatographic peaks were in good shape, and there was no interference from other endogenous substances. The regression equation of PG was y = 0.584781x − 0.47063, and the *r* ^2^ and range of the linear were 0.9929 and 0.001–40 μg mL^−1^, respectively. LLOQ was 0.5 ng mL^−1^. The RSD of the intraday and interday precision was less than 15%, and the accuracy was between 80% and 120% (Supplementary Table S11). Results showed that the RSD of matrix effect and extraction recovery were all less than 13.12% and 12.81% (Supplementary Table S12). The stability of PG was less than 12.83% (Supplementary Table S13 **)**. It was shown that the established method complies with the relevant regulations of pharmacokinetics.

#### 3.5.2 Pharmacokinetic Analysis

**FIGURE 6:**
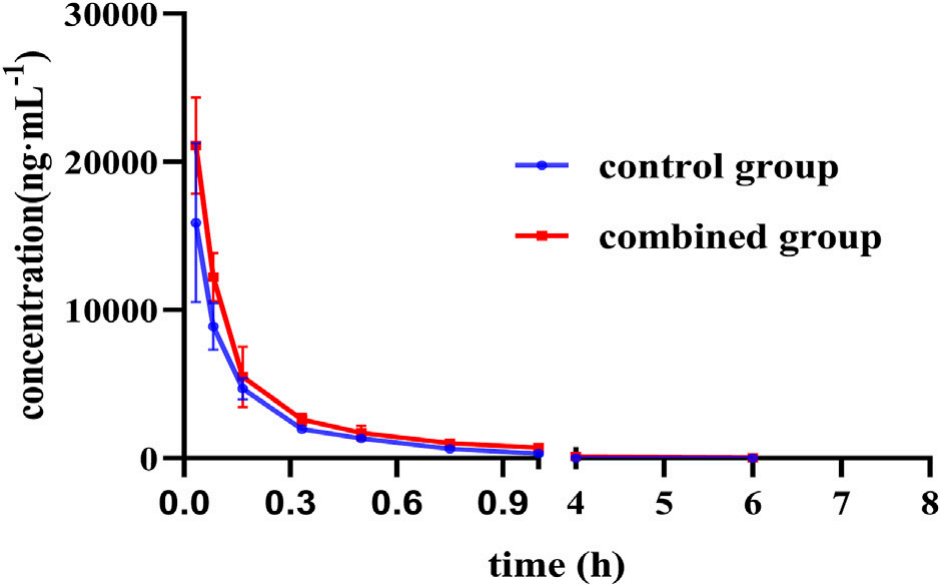
Mean plasma concentration–time curve of PG in different groups (*n* = 8).

The average blood concentration–time curve of PG is shown in Figure 6. Pharmacokinetic parameters were calculated under a noncompartmental model using Drug and Statistics 1.0 (DAS 1.0) software. The results showed that T_1/2_ (*p* < 0.05) was prolonged and the clearance of PG was significantly decreased (*p* < 0.05). AUC _0-t_ and AUC _0-∞_ (*p* < 0.05) of PG were significantly increased in the combined group **(** Table 2 **)**, and there were significant differences between the two groups of data.

**TABLE 2:** Pharmacokinetic parameters of PG alone and in combination with GAO (*n* = 8).

Group	AUC _0-t_ (μg·h·L^−1^)	AUC_0-∞_ (μg·h· L^−1^)	t_1/2_ (h)	CL (L·min^−1^·kg^−1^)
Control	3306.490 ± 516.846	3317.058 ± 524.878	1.013 ± 0.409	2.774 ± 0.441
Combined	4895.574 ± 606.408*	5005.963 ± 685.510*	1.739 ± 0.802*	1.827 ± 0.240*

## 4 Discussion

Mathematical models that combine *in vitro* findings and *in vivo* pharmacokinetic data play an important role in investigating the DDI potential of a drug. The results of *in vitro* experiment showed that GAO was an inhibitor of UGTs and PG was a substrate of UGTs. The *in vivo* results showed that the pharmacokinetic parameters, including T_1/2_, CL, AUC _0-t_, and AUC _0-∞_ of PG, have significant differences between the control group and the combined group. It is validated that the combination of the two drugs would change the pharmacokinetics of PG.

The quantitative experiment of GAO showed that the content of flavonoids was relatively high. After administration of GAO, the total flavonoid glycosides will be hydrolyzed into flavonoid aglycones, including quercetin, isorhamnetin, and kaempferol. The inhibitory effects of flavonoids on UGT1A enzymes have been reported in the literature (D'Andrea et al., 2005). Literature showed that quercetin was a moderate inhibitor of UGT1A1 and UGT1A3, a weak inhibitor of UGT1A6, and a strong inhibitor of UGT1A9 (Zhang et al., 2021). Both luteolin and quercetin inhibited human hepatic UGT1A1 and UGT1A4 in an inhibition study *in vitro* (Cao et al., 2017). In addition, amentoflavone displayed strong inhibition toward most human UGTs, including UGT1A1, 1A3, 1A4, 1A6, 1A7, 1A8, and 1A9 (Lv et al., 2018). PG was a substrate of UGTs; for substrates metabolized mainly through glucuronidation, modulation of UGT activities could lead to significant effects on pharmacokinetics (Kiang et al., 2005). It is speculated that a large part of the reason for the DDI between GAO and PG from the regulation of UGTs by GAO, i.e., the flavonoids in GAO are likely to increase the exposure level of PG *in vivo* by inhibiting the activity of UGTs.

In previous studies, kaempferol exhibited a remarkable inhibition of the P-glycoprotein-mediated efflux of ritonavir. Literature showed that kaempferol was also an inhibitor of breast cancer resistance protein (An et al., 2011; Palatini and De Martin, 2016). Therefore, repeated administration of GAO will alter the pharmacokinetic behavior of PG, which is likely to be mediated by transporters in addition to UGT. In addition, some further in-depth research is needed, such as elucidation on the exact components of ginkgo flavonoids, ginkgolides, and armillaria, which contribute to the inhibitory effect. Both transporter and SULT effects also need to be fully investigated.

## 5 Conclusion

An UHPLC/Q-Orbitrap MS method was established to characterize the chemical constituents in GAO. A total of 62 compounds were identified or tentatively identified. Furthermore, nine main compounds were determined using UPLC-QQQ-MS of GAO; the content of flavonoids and ginkgolides was higher than those of the others. The results of *in vitro* liver microsomes incubation experiment showed that GAO had a significant inhibitory effect on UGT1A9 and UGT2B7. PG was mainly metabolized using UGT and SULT *in vitro*. PK DDI studies showed that T_1/2_ was prolonged and the clearance of PG was significantly decreased, and AUC _0-t_ and AUC _0-∞_ of PG were significantly increased when repeating GAO.

## Data Availability

The original contributions presented in the study are included in the article/Supplementary Material, further inquiries can be directed to the corresponding authors.
