# Supplementary material for: Studies on Chemical Characterization of Ginkgo Amillaria Oral Solution and Its Drug–Drug Interaction With Piceatannol 3′-O-β-D-Glucopyranoside for Injection
Source: Front Pharmacol. 2022 Jul 19;13:932646. doi: 10.3389/fphar.2022.932646 (PMC9344054; doi:10.3389/fphar.2022.932646)
Supplement: Supplementary file 1 [file DataSheet1.doc]

**SUPPORTING INFORMATION**

**Studies on chemical characterization of Ginkgo** **Amillaria oral solution and its drug-drug interaction with piceatannol 3'-*O*-*β*-D-glucopyranoside for injection**

Zhenyan Yu^1^, Xiaohan Hu^1^, Lin Zhou^1^, Huliang Chen^1^, Yanchao Xing^1^, Chunyue Han^1^, Hui Ding^1^, Lifeng Han^1^, Guixiang Pan^2,*^, Zhifei Fu^1,*^

# Affiliations

*^1^* *State Key Laboratory of Component-based Chinese Medicine, Tianjin University of Traditional Chinese Medicine, 10 Poyanghu Road, Jinghai, Tianjin 301617, P. R. China*

*^2^* *Second Affiliated hospital of Tianjin University of Traditional Chinese Medicine, Tianjin, 300250, China*


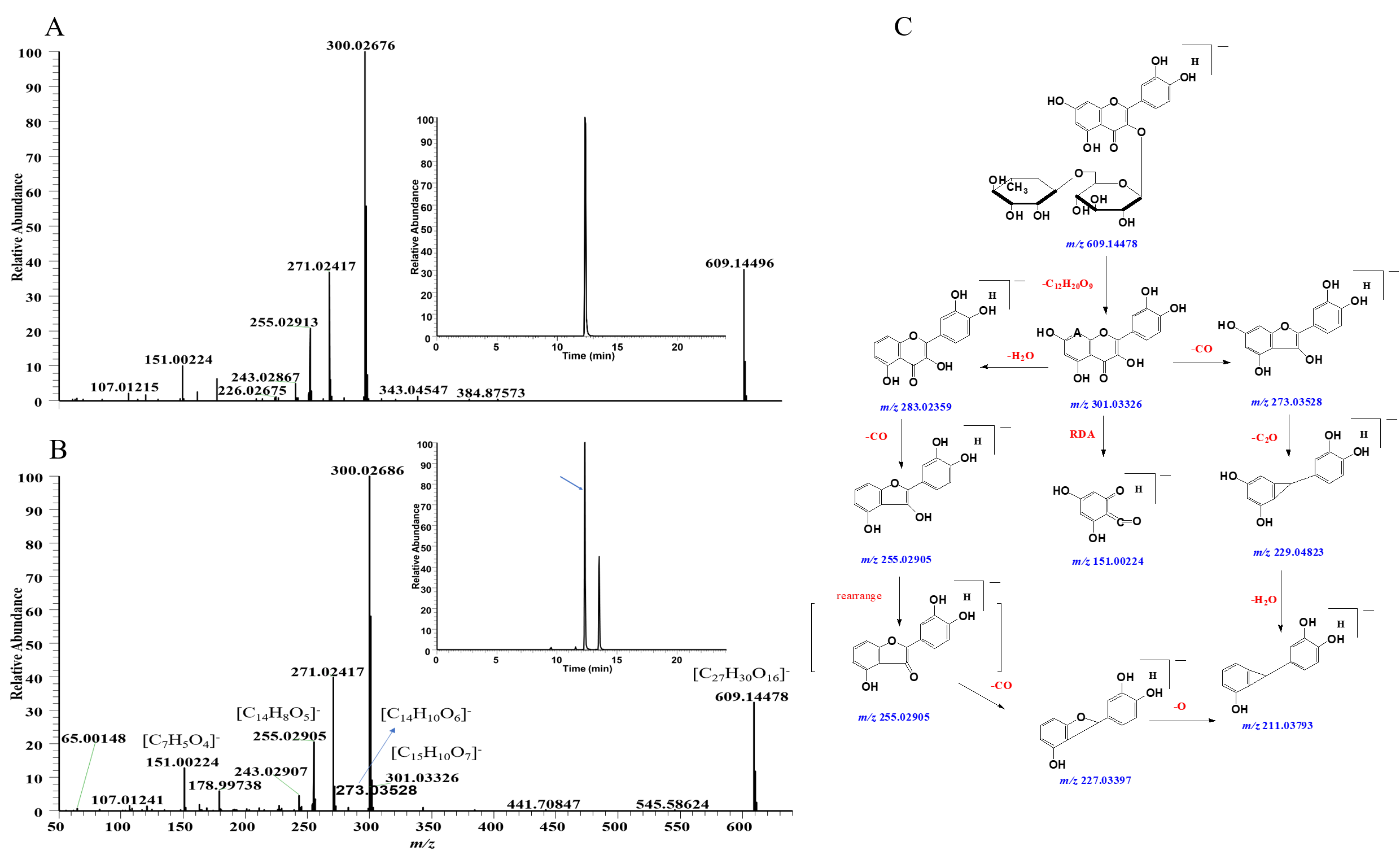


**Fig. S1.** The MS/MS spectra of rutin (A) and GAO (B) and possible fragmentation pathways (C) of **Comp. 40** (**Table S2**)


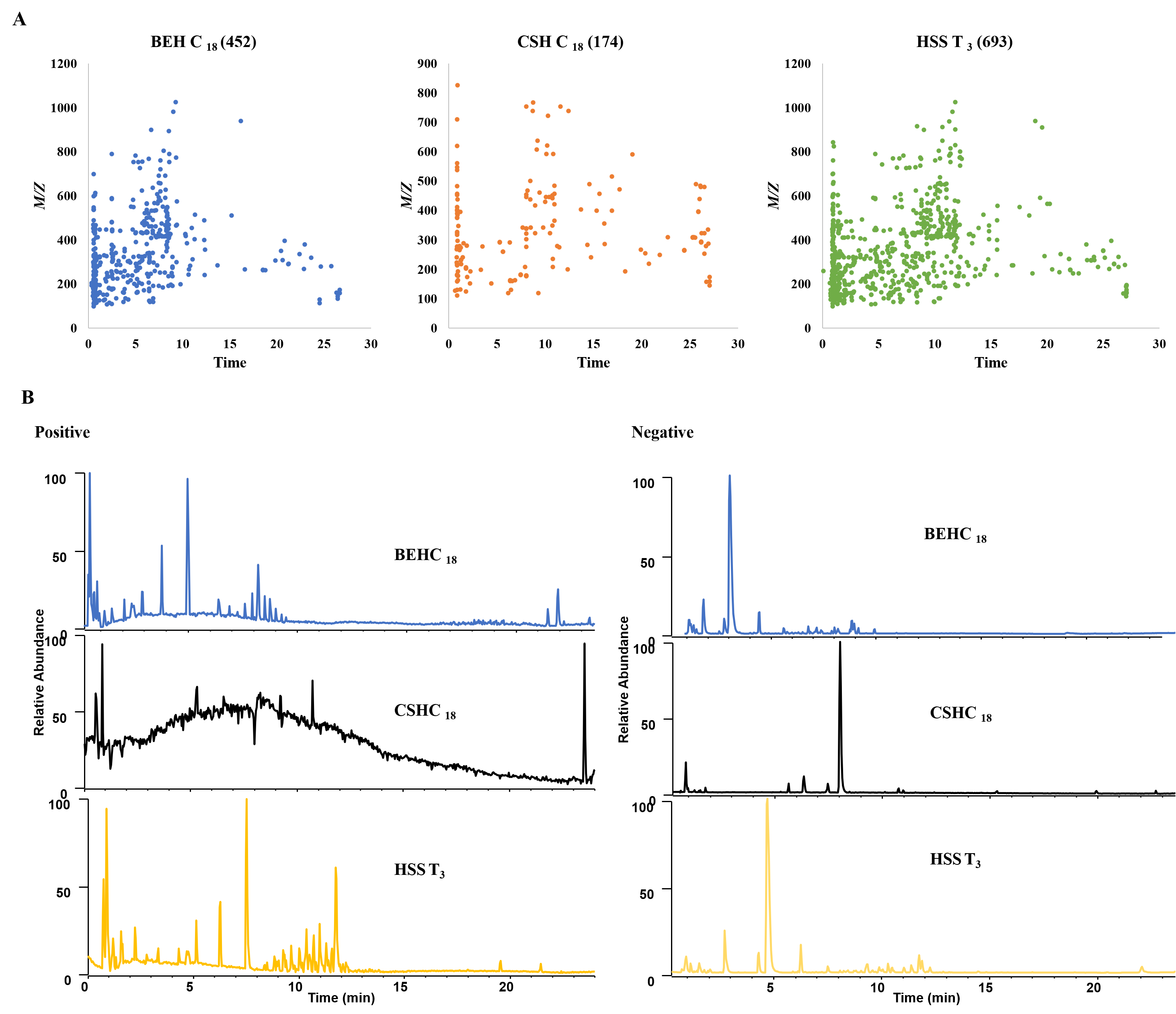


**Fig. S2** The number of chromatographic peaks of sample detected by the three reversed-phase chromatographic columns in the negative ion mode (A); the total ion chromatography of the GAO sample in the positive and negative ion modes of the three chromatographic columns (B)


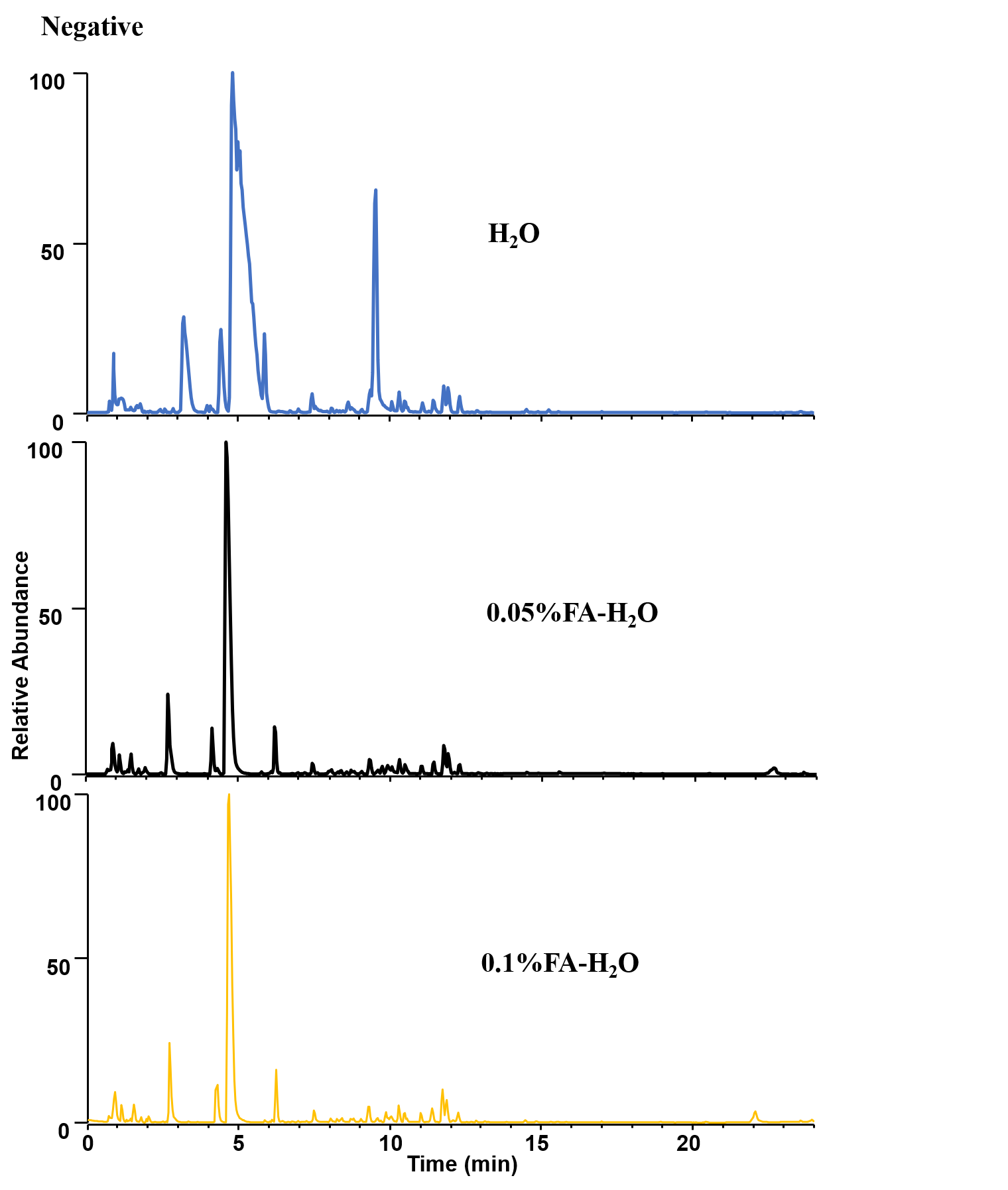


**Fig. S3** Total ion chromatography of different additives in water phase


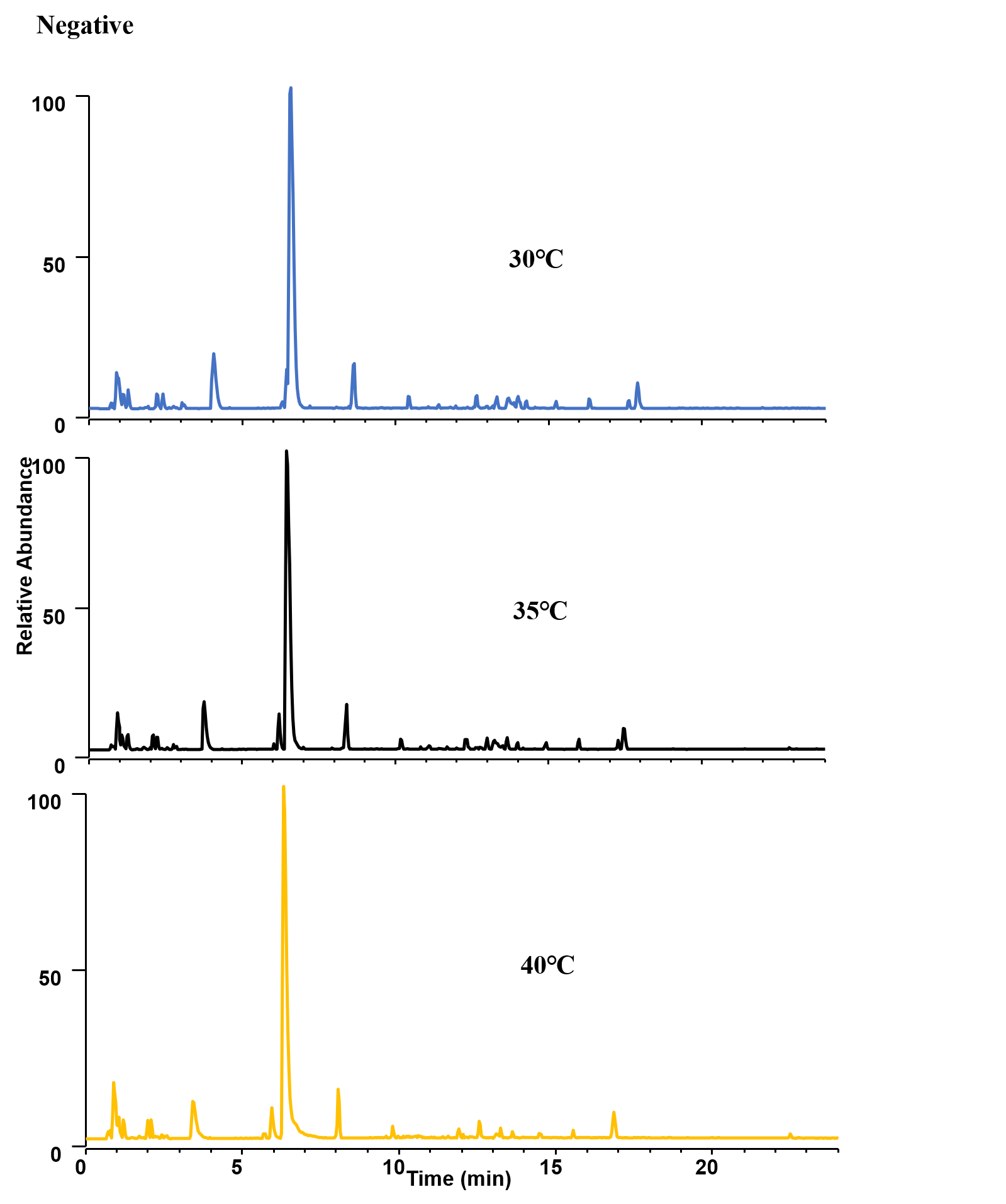


**Fig. S4** Total ion chromatography of different column temperatures

**Fig. S5** The effect of spray voltage (A), capillary temperature (B) and auxiliary gas temperature (C) in negative ion mode of Q-Orbitrap mass spectrometry


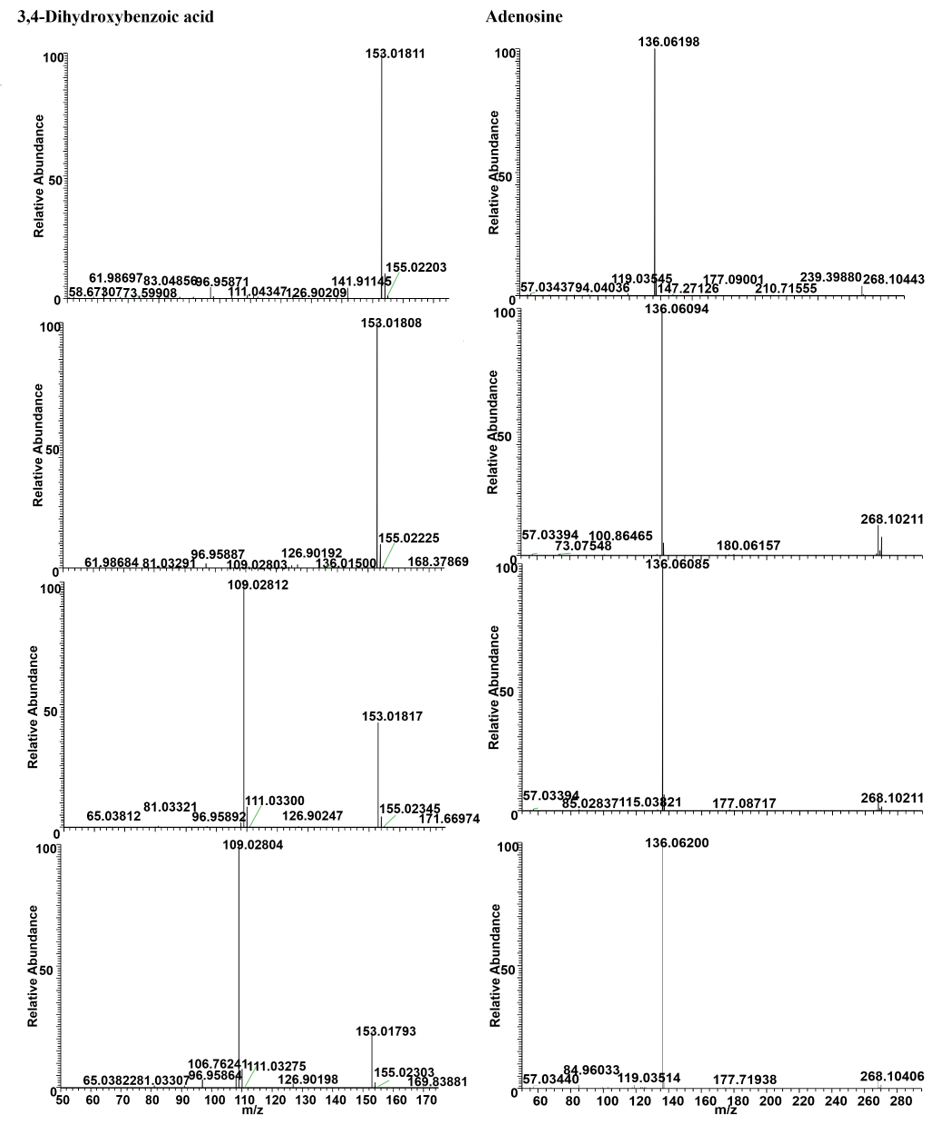

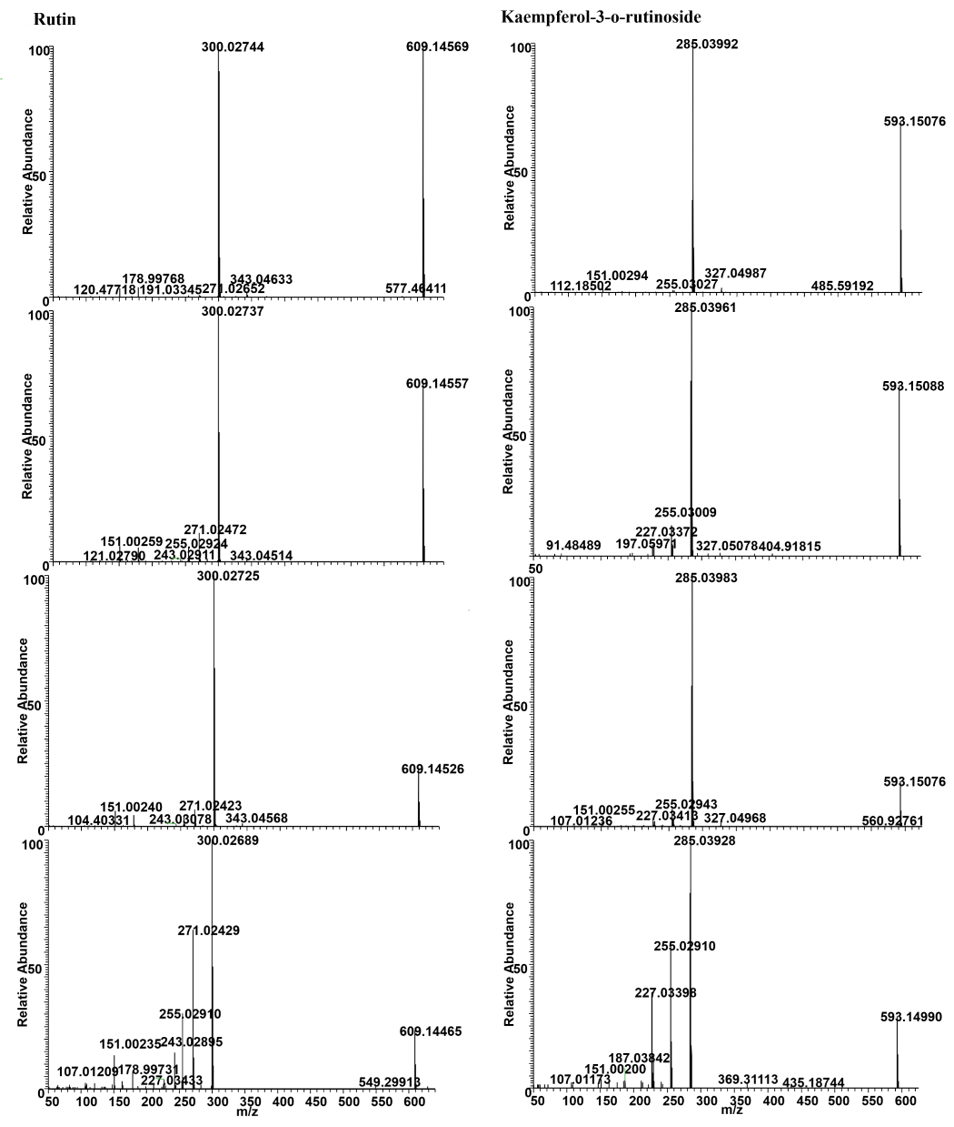

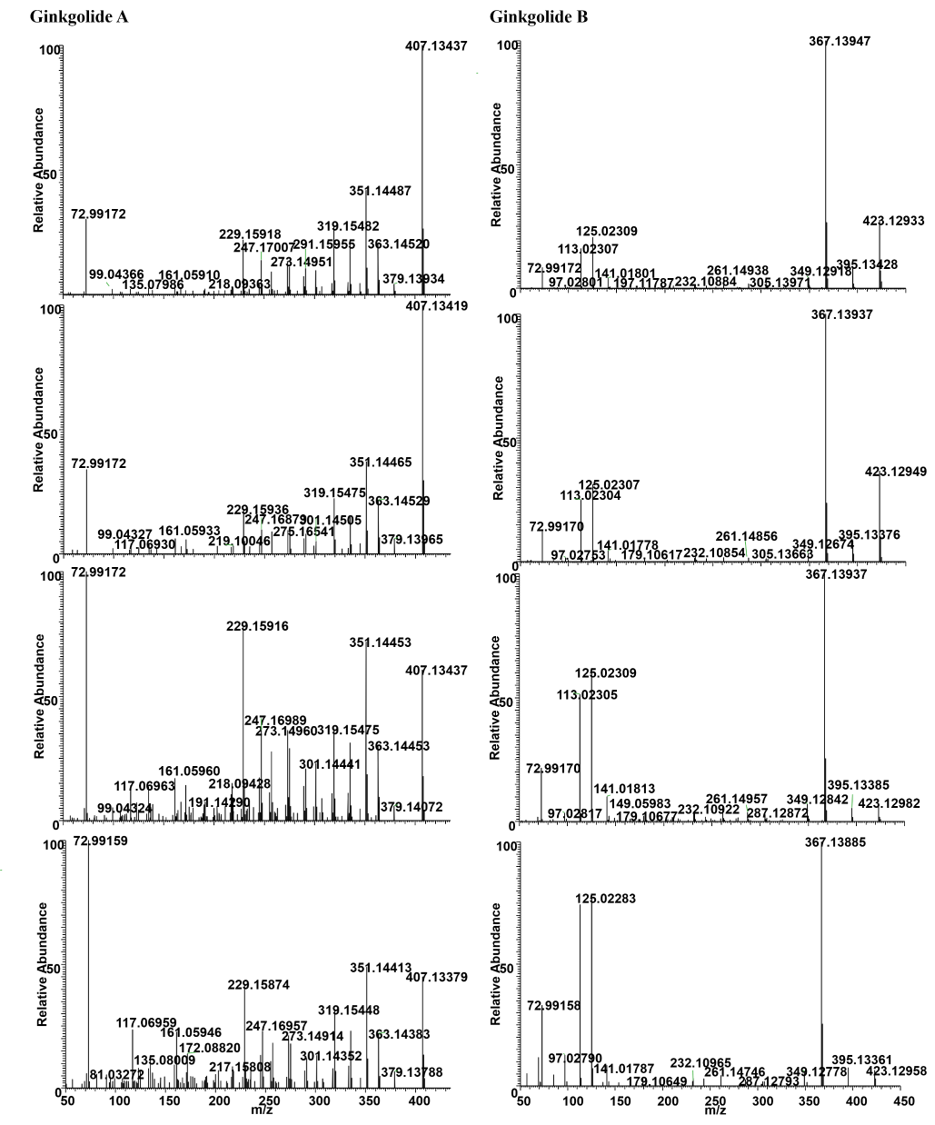


**Fig. S6** Optimized normalized collision energies in positive and negative ion modes of Q-Orbitrap mass spectrometer (set four normalized collision energies from top to bottom: 10/20/30 V; 10/20/40 V; 20/ 30/40 V; 20/40/60 V)


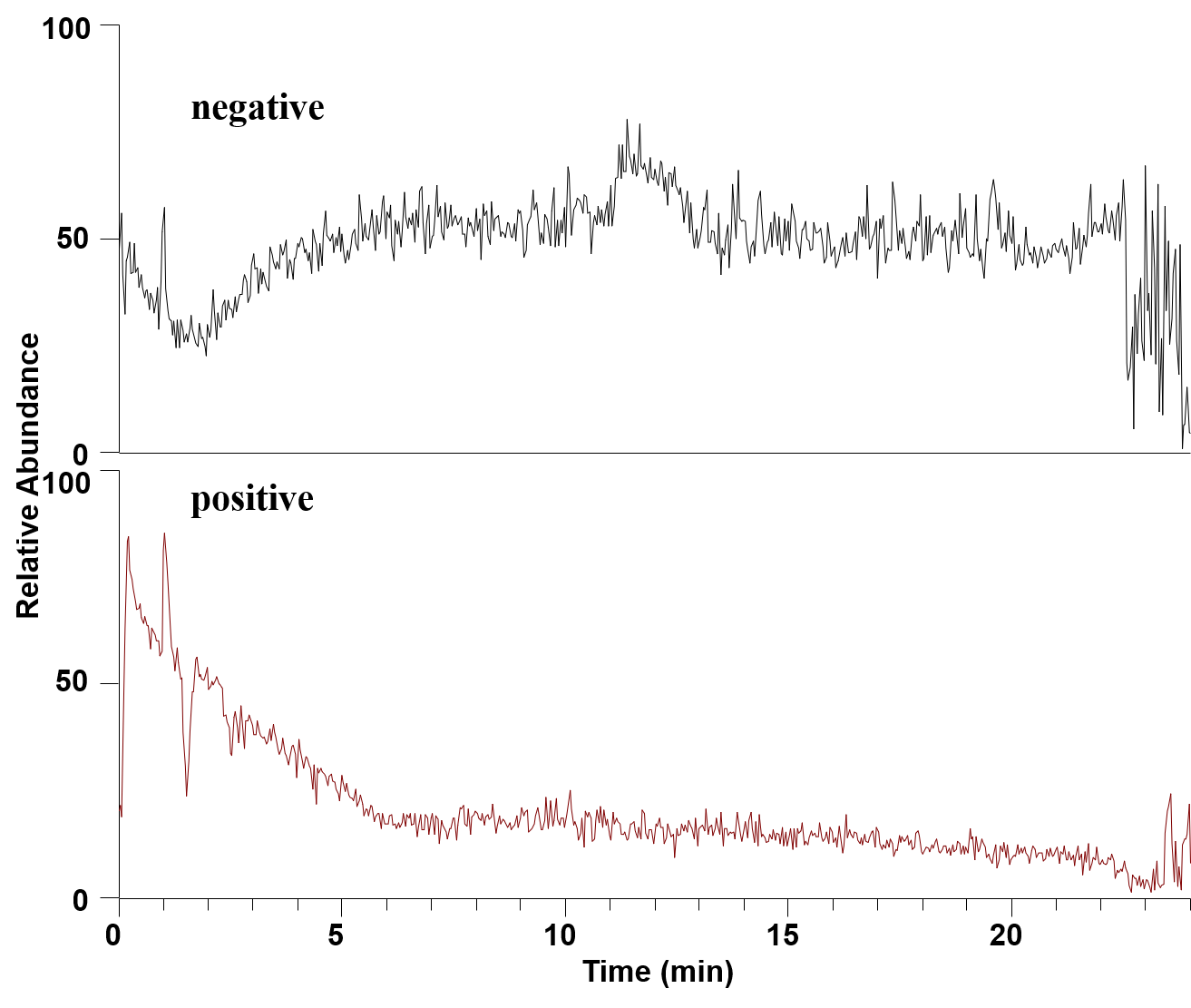


**Fig. S7** The blank solution control of GAO


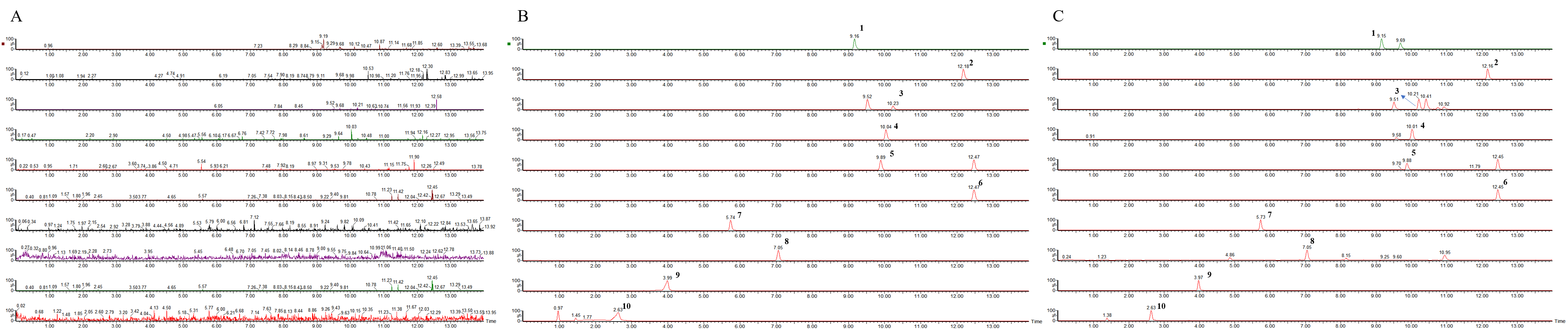


**Fig. S8** A: MRM diagram of nine compounds in blank solvent; B: MRM diagram of nine compounds in mixed standard solution; C: MRM diagram of nine compounds in GAO sample (**1**: rutin; **2**: Linarin; **3**: astragalin; **4**: ginkgolide C; **5**: ginkgolide J; **6**: ginkgolide A; **7**: 3,4-dihydroxybenzoic acid; **8**: *p*-hydroxybenzoic acid; **9**: guanosine; **10**: uridine)

**Fig. S9** The content of nine compounds in 13 batches of GAO


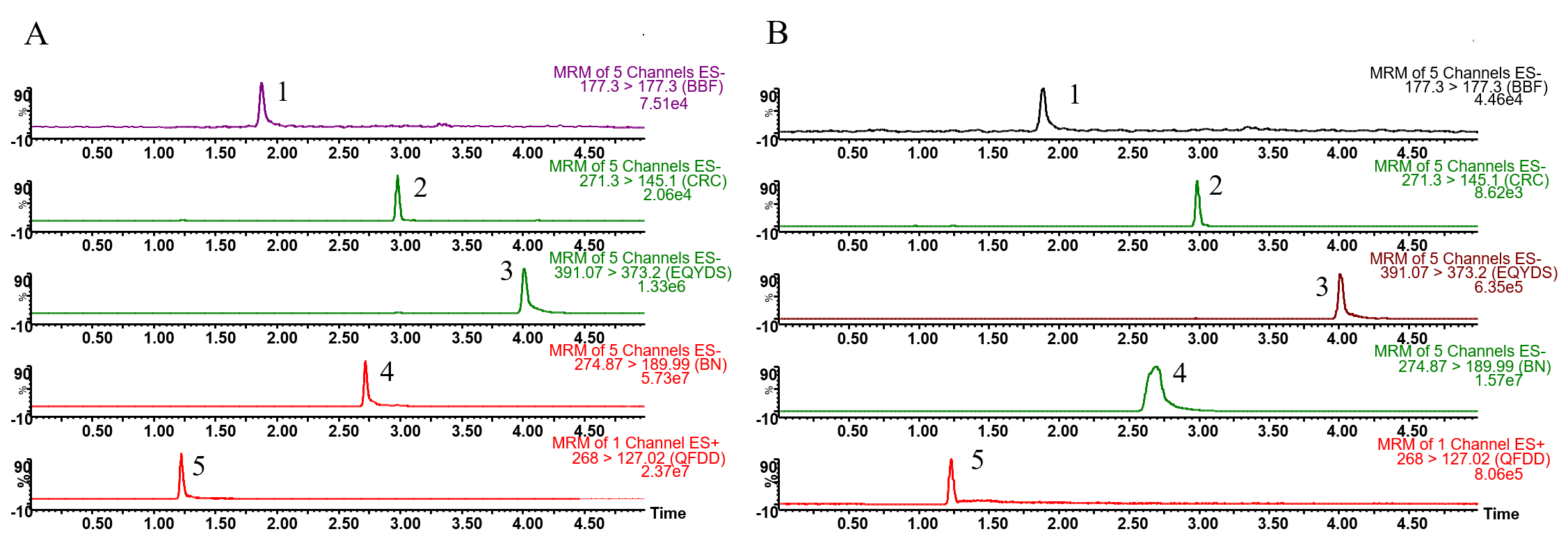


**Fig. S10** A: MRM diagram of five compounds in mixed standard solution; B: MRM diagram of six compounds in sample (**1**: 2,6-diisopropylphenol; **2**: estradiol; **3**: chenodeoxycholic acid; **4**: chlorpropamide; **5**: zidovudine)


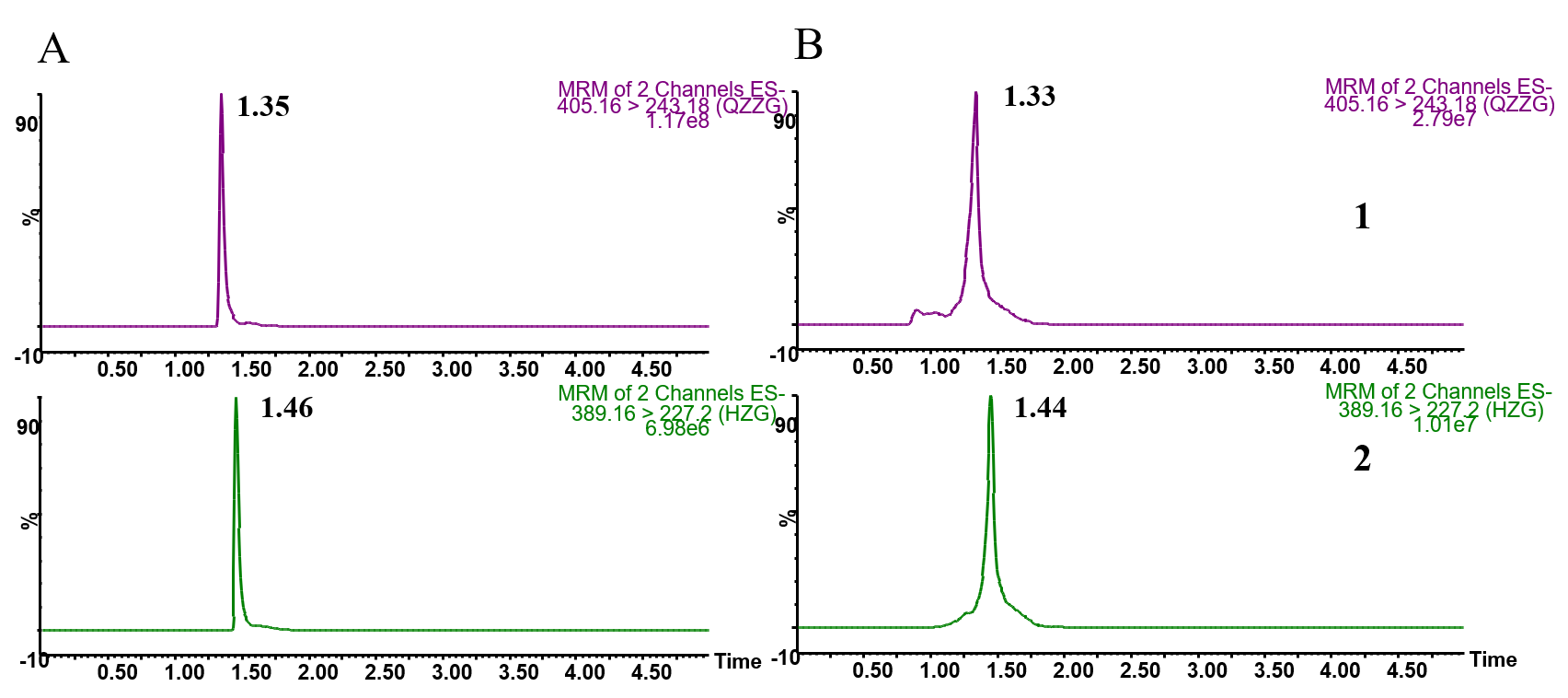


**Fig. S11** MRM diagram of two compounds in mixed standard solution (A); MRM diagram of two compounds in sample (B) (**1**: PG; **2**: polydatin)


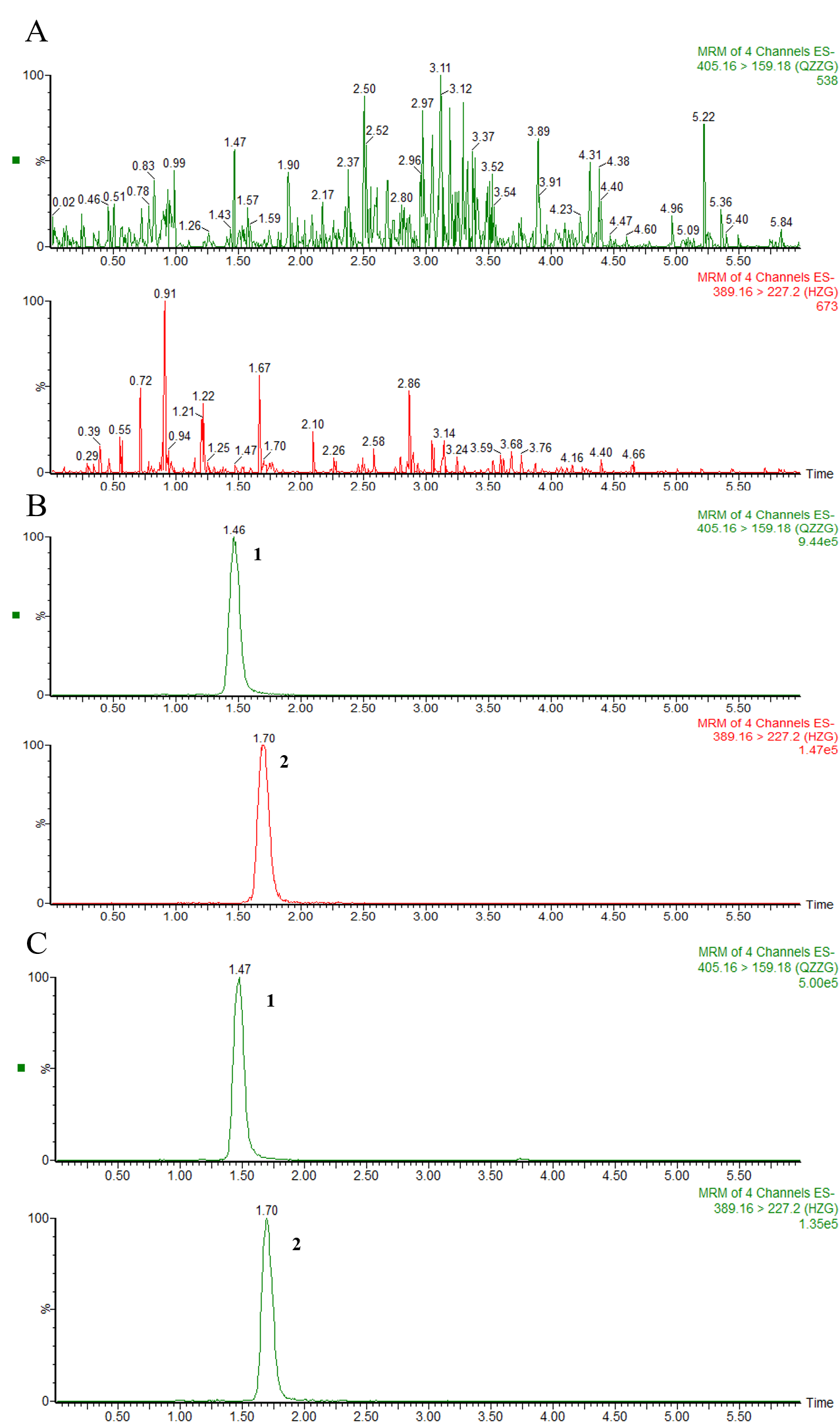


**Fig. S12** MRM diagram of blank plasma (A); MRM diagram of blank plasma added with a certain concentration of reference substance (B); After oral administration to rats MRM map of 20 min plasma samples (C) (**1**: PG; **2**: polydatin)

**Table S1** Transition and mass spectrometry parameters of seven compounds

| Comp. | Detect  mode | Precursor  ions (*m/z*) | Product  ions (*m/z*) | Cone voltage (V) | Collision energy (V) |
| --- | --- | --- | --- | --- | --- |
| 2,6-diisopropylphenol | ESI- | 177.30 | 177.30 | 120 | 0 |
| estradiol | ESI- | 271.30 | 145.10 | 150 | 40 |
| chenodeoxycholic acid | ESI- | 391.07 | 373.29 | 4 | 24 |
| chlorpropamide (IS) | ESI- | 274.87 | 189.99 | 66 | 18 |
| zidovudine | ESI+ | 268.00 | 127.02 | 6 | 12 |
| PG | ESI- | 405.16 | 243.18 | 80 | 30 |
| polydatin (IS) | ESI- | 389.16 | 227.20 | 84 | 20 |

**Table S2** Identification of compounds in GAO by UHPLC-/ESI-Q-Orbitrap MS method in ESI^+^/ ESI^-^ mode

| **Comp.** | **RT (min)** | ***m/z***  **(pos/neg)** | **Formula** | **RDB** | ***δ* ppm** | **MS/MS**  **(*m/z*)** | **Identification** | **Category** |
| --- | --- | --- | --- | --- | --- | --- | --- | --- |
| **1** | 0.85 | 175.11891/+ | C_6_H_14_N_4_O_2_ | 1.5 | -0.241 | 158.09233,146.96121,135.94539,130.09752,125.00265,116.07087,70.06583,60.05642,55.93521 | L-arginine ^*^ | amino acid |
| **2** | 0.86 | 132.02876/- | C_4_H_7_NO_4_ | 2.5 | -0.374 | 115.00219,88.03893,71.01241,134.04567,89.04215,87.04375,116.00581,114.01819 | L-aspartic acid ^*^ | amino acid |
| **3** | 0.91 | 165.07573/+ | C_6_H_12_O_5_ | 0.5 | -0.121 | 167.0556,147.06519,129.05472,111.04430,99.04444,85.02893,83.04971,81.03402,74.02428,69.03419,61.02919,57.03430 | L-rhamnose ^a^ | amino acid |
| **4** | 0.93 | 181.07060/- | C_6_H_14_O_6_ | 0.5 | -0.357 | 163.05983,143.86371,119.03346,101.02293,96.96812,89.02296,71.01241,59.01244,73.02807,85.02807 | D-mannite ^a^ | sugar alcohol |
| **5** | 0.98 | 112.05075/- | C_4_H_5_N_3_O | 3.5 | 0.212 | 113.05403,131.97417,96.00917,95.02438,72.93774,69.04538,57.03415 | cytosine ^*^ | pyrimidine |
| **6** | 1.03 | 191.05493/- | C_7_H_12_O_6_ | 2.5 | 1.097 | 102.94727,85.02801,61.98683,143.86372,146.93716,211.89561,71.01233,79.95567 | D-quinic acid ^*^ | cyclohexanecarboxylic acid |

Continued from **Table S2**

| **Comp.** | **RT (min)** | ***m/z***  **(pos/neg)** | **Formula** | **RDB** | ***δ* ppm** | **MS/MS**  **(*m/z*)** | **Identification** | **Category** |
| --- | --- | --- | --- | --- | --- | --- | --- | --- |
| **7** | 1.07 | 503.16110/- | C_18_H_32_O_16_ | 3.5 | 0.872 | 341.10867,323.09662,179.05495,161.04425,101.02291,89.02291,71.01234 | xylosucrose ^a^ | trisaccharide |
| **8** | 1.17 | 665.21490/- | C_24_H_42_O_21_ | 4.5 | 1.416 | 587.18201,545.16962,503.16357,383.11884,341.10818,263.07623,221.06577,179.05505,161.04425,143.03351,113.02287,101.02291,71.01234 | glycogen ^a^ | polysaccharide |
| **9** | 1.31 | 281.08701/- | C_10_H_18_O_9_ | 2.5 | 0.301 | 249.06125,205.07077,221.06680,189.03969,129.01749,119.03323,113.02284,101.02277,89.02285,85.02794,71.01228,59.01237,87.00719 | xylobiose ^a^ | disaccharide |
| **10** | 1.33 | 118.08640/+ | C_5_H_11_NO_2_ | 0.5 | 1.227 | 72.08141,73.08472,70.06573,55.05503,53.03937,90.94813,97.00797,108.95855,120.02370 | valine ^*^ | amino acid |
| **11** | 1.35 | 177.03906/- | C_6_H_10_O_6_ | 2.5 | -0.304 | 159.0284,161.04411,141.01775,129.01753,131.03329,115.03851,101.02264,85.02792,87.04364,89.02276,72.99153,71.01226,59.01235 | D- (+)-gluconic acid δ-lactone ^a^ | polyhydroxy acid |

Continued from **Table S2**

| **Comp.** | **RT (min)** | ***m/z***  **(pos/neg)** | **Formula** | **RDB** | ***δ* ppm** | **MS/MS**  **(*m/z*)** | **Identification** | **Category** |
| --- | --- | --- | --- | --- | --- | --- | --- | --- |
| **12** | 1.39 | 173.00784/- | C_6_H_6_O_6_ | 4.5 | -0.224 | 174.95515,173.00784,172.06017,171.01872 | trans-aconitic acid ^*^ | carboxylic acid |
| **13** | 1.53 | 343.12378/- | C_12_H_24_O_11_ | 1.5 | 0.851 | 253.092,232.97408,211.45732,178.91844,158.97304,121.62246,101.02267,89.02280,71.01199,59.01226 | melibiitol ^a^ | sugar alcohol |
| **14** | 1.55 | 136.06158/+ | C_5_H_5_N_5_ | 5.5 | -1.410 | 139.13219,119.03520,107.08541,95.08569,90.94794,81.07041,74.95319,67.05473,56.94286 | adenine ^*^ | purine |
| **15** | 1.58 | 244.09279/+ | C_9_H_13_N_3_O_5_ | 4.5 | -0.029 | 221.98270,209.05524,191.04352,151.01793,137.934,125.03461,112.05081,102.94827,95.02437 | cytidine ^*^ | nucleosides |
| **16** | 1.62 | 305.13397/+ | C_12_H_20_N_2_O_7_ | 3.5 | -1.171 | 287.12271,269.11282,251.10165,241.11865,187.08727,157.07610 | 2'-deoxymugineic acid ^a^ | non-protein amino acids |
| **17** | 1.63 | 113.03456/+ | C_4_H_4_N_2_O_2_ | 3.5 | 0.054 | 114.03798,112.05058,108.95836,103.01042,96.00822,95.02416,90.94798,86.06026,78.51030,72.93761,70.02925,67.05488 | uracil ^*^ | pyrimidine |
| **18** | 1.88 | 123.05520/+ | C_6_H_6_N_2_O | 4.5 | -0.089 | 125.09602,124.07556,121.06474,112.03938,96.04467,95.04942,93.07018,81.07028,80.04993,79.05465 | niacinamide ^a^ | amides |

Continued from **Table S2**

| **Comp.** | **RT (min)** | ***m/z***  **(pos/neg)** | **Formula** | **RDB** | ***δ* ppm** | **MS/MS**  **(*m/z*)** | **Identification** | **Category** |
| --- | --- | --- | --- | --- | --- | --- | --- | --- |
| **19** | 2.83 | 245.07681/+ | C_9_H_12_N_2_O_6_ | 4.5 | -0.011 | 133.04959,113.03479,96.00841,70.02937,57.03434 | uridine ^*^ | nucleosides |
| **20** | 2.84 | 132.10193/+ | C_6_H_13_NO_2_ | 0.5 | 0.188 | 113.96391,108.95863,93.03712,87.10023,86.09694,75.02667,72.93777,69.07069,57.03425 | L-leucine ^*^ | amino acid |
| **21** | 2.87 | 182.08105/+ | C_9_H_11_NO_3_ | 4.5 | -0.120 | 165.0547,154.99107,159.96910,136.07574,123.04428,113.96397,97.96931,72.93778,56.94304 | L-tyrosine ^*^ | amino acid |
| **22** | 3.98 | 169.01308/- | C_7_H_6_O_5_ | 5.5 | -0.413 | 171.01851,126.87959,125.02296,122.89276,97.02802,81.03304,69.03303,103.91872 | gallic acid ^*^ | polyphenols |
| **23** | 4.00 | 268.10367/+ | C_10_H_13_N_5_O_4_ | 6.5 | -1.344 | 136.06175, 133.04932,119.03522,97.02877,85.02895,57.03426 | adenosine ^*^ | nucleosides |
| **24** | 4.27 | 284.09909/+ | C_10_H_13_ N_5_O_5_ | 6.5 | 0.510 | 223.52647,189.84668,152.05663,143.24721,135.03000,110.03531,85.02886,73.02882 | guanosine ^*^ | nucleosides |
| **25** | 4.30 | 152.05667/+ | C_5_H_5_N_5_O | 5.5 | -0.108 | 146.02966,135.11687,129.97899,111.96857,109.10147,97.06519,95.08595,69.07057,59.04992 | guanine ^*^ | purine |

Continued from **Table S2**

| **Comp.** | **RT (min)** | | ***m/z***  **(pos/neg)** | **Formula** | **RDB** | ***δ* ppm** | **MS/MS**  **(*m/z*)** | **Identification** | **Category** |
| --- | --- | --- | --- | --- | --- | --- | --- | --- | --- |
| **26** | 4.48 | | 161.98523/- | C_4_H_5_NO_4_S | 3.5 | -2.005 | 82.02835,77.96368,101.92352,85.02752,61.98668,103.91873,143.86386,119.04834 | 6-methyl-1,2,3-oxathiazin-4(3H)-one 2,2-dioxide potassium salt ^b^ | organic synthetic salt |
| **27** | 4.96 | 166.08609/+ | | C_9_H_11_NO_2_ | 4.5 | -0.994 | 131.04922,122.08768,120.08092,118.03513,120.08092,84.96021,103.05453,93.07033 | L-phenylalanine ^a^ | amino acid |
| **28** | 5.19 | | 127.03900/+ | C_6_H_6_O_3_ | 3.5 | 0.029 | 118.03508,109.02864,99.04432,93.03727,81.03402,79.05470,57.03423,53.03938 | 5-hydroxymethyl-2-furaldehyde ^*^ | furfural compounds |
| **29** | 5.96 | | 153.01808/- | C_7_H_6_O_4_ | 5.5 | -0.155 | 141.91061,126.90173,109.02810,106.99152,81.03319,69.03323,61.98695 | 3,4-dihydroxybenzoic acid ^*^ | polyphenols |
| **30** | 6.23 | | 127.03880/+ | C_6_H_6_O_3_ | 3.5 | -0.171 | 129.04367,118.03423,109.02845,100.07529,84.96002,81.03407,71.04966,67.93530,53.03914 | 1,2,3-trihydroxybenzene ^a^ | phenolic compound |

Continued from **Table S2**

| **Comp.** | **RT (min)** | ***m/z***  **(pos/neg)** | **Formula** | **RDB** | ***δ* ppm** | **MS/MS**  **(*m/z*)** | **Identification** | **Category** |
| --- | --- | --- | --- | --- | --- | --- | --- | --- |
| **31** | 6.37 | 178.05318/- | C_6_H_13_NO_3_S | 1.5 | -0.340 | 193.4529,170.97818,164.26024,111.50742,81.95174,79.95589,72.06903,58.63231 | cyclamic acid ^b^ | sweetener |
| **32** | 7.51 | 139.03868/- | C_7_H_6_O_3_ | 5.5 | 1.097 | 98.9846,87.0445,71.0691,70.0658,69.0341,59.0737,55.0549 | *p*-hydroxybenzoic acid ^*^ | phenol derivative |
| **33** | 8.05 | 289.07123/- | C_15_H_14_O_6_ | 9.5 | 1.956 | 245.08119,221.08112,203.07021,179.03305,151.03883,125.02289,125.02289,109.02799 | epicatechin ^*^ | flavonoids |
| **34** | 8.35 | 239.05524/- | C_11_H_12_O_6_ | 6.5 | 0.943 | 221.04413,203.28990,195.06470,179.03375,149.05939,133.06430,121.02827,107.04870,87.00729 | (1R,6R)-6-hydroxy-2-succinylcyclohexa-2,4-diene-1-carboxylate ^a^ | carboxylic acid |
| **35** | 9.65 | 289.07120/- | C_15_H_14_O_6_ | 9.5 | 1.852 | 245.08138,203.07014,151.03850,125.02261,109.02800,205.04897,97.02744,91.02034 | catechin^*^ | flavonoids |

Continued from **Table S2**

| **Comp.** | **RT (min)** | ***m/z***  **(pos/neg)** | **Formula** | **RDB** | ***δ* ppm** | **MS/MS**  **(*m/z*)** | **Identification** | **Category** |
| --- | --- | --- | --- | --- | --- | --- | --- | --- |
| **36** | 10.09 | 295.12833/+ | C_14_H_18_N_2_O_5_ | 6.5 | -1.756 | 277.11798,260.09171,235.10760,200.07050,180.10185,175.08662, 120.08096,88.03981 | (3R)-3-amino-4-methoxy-4-oxobutanoic acid ^a^ | amino acid derivatives |
| **37** | 10.78 | 209.04453/- | C_10_H_10_O_5_ | 6.5 | 0.383 | 182.95337,162.98164,154.95824,136.01523,121.02797,103.91851,72.99153,61.98667 | 5-hydroxyferulate ^a^ | hydroxycinnamic acid |
| **38** | 10.89 | 211.14363/+ | C_11_H_18_N_2_O_2_ | 3.5 | -0.474 | 183.14879,138.12743,138.12743,114.09139,107.05236,98.06023,93.03688,86.09675,81.07021,75.02650,70.06564 | L, L-cyclo (leucylprolyl) ^a^ | amino acid derivatives |
| **39** | 11.35 | 343.10266/- | C_15_H_20_O_9_ | 6.5 | 0.879 | 325.09204,299.11307,301.11823,281.10278,237.11220,219.10135,181.12213,193.12218,165.12668,163.11142 | 4',6'-dihydroxy-2'-methoxyacetophenone 6'-glucoside ^a^ | glycoside |
| **40** | 12.24 | 609.14539/- | C_27_H_30_O_16_ | 13.5 | 1.801 | 300.02704,271.02441,255.02914,243.02911,178.99751,151.00230,121.02753 | rutin ^*^ | flavone glycosides |

Continued from **Table S2**

| **Comp.** | **RT (min)** | ***m/z***  **(pos/neg)** | **Formula** | **RDB** | ***δ* ppm** | **MS/MS**  **(*m/z*)** | **Identification** | **Category** |
| --- | --- | --- | --- | --- | --- | --- | --- | --- |
| **41** | 12.36 | 901.23877/- | C_42_H_46_O_22_ | 20.5 | -1.031 | 739.18597,593.14850,446.08389,413.08783,339.18137,284.03174,255.02904,227.03366,213.05524,145.02800,91.86799 | kaempferol 3-[2''-(6'''-coumaroylglucosyl)-rhamnoside] 7-glucoside ^a^ | flavone glycosides |
| **42** | 12.59 | 423.12921/- | C_20_H_24_O_10_ | 9.5 | 0.088 | 405.11829,379.13840,349.09207,305.10342,261.11270,233.11761,209.04449,177.05460,147.04384,121.02805,81.03278,73.02799 | ginkgolide J ^*^ | ginkgolide |
| **43** | 12.71 | 465.10153/+ | C_21_H_20_O_12_ | 11.5 | -2.628 | 303.04959,85.02892,97.02878,127.03899,127.03899,69.03419,81.03408,91.03904,137.02318,229.04982,257.04440 | isoquercitrin ^b^ | flavonoids |
| **44** | 12.81 | 447.09235/- | C_21_H_20_O_11_ | 12.5 | 0.363 | 139.03868,108.89821,94.91542,93.03308 | astragalin ^*^ | flavonoids |
| **45** | 12.82 | 439.12387/- | C_20_H_24_O_11_ | 9.5 | -0.166 | 411.12769,383.13394,365.12283,321.13248,259.13263,231.13914,196.10709,141.01741,125.02293,113.02284,97.02771,72.99162 | ginkgolide C ^*^ | ginkgolide |

Continued from **Table S2**

| **Comp.** | **RT (min)** | ***m/z***  **(pos/neg)** | **Formula** | **RDB** | ***δ* ppm** | **MS/MS**  **(*m/z*)** | **Identification** | **Category** |
| --- | --- | --- | --- | --- | --- | --- | --- | --- |
| **46** | 13.39 | 611.16052/+ | C_27_H_30_O_16_ | 12.5 | -0.231 | 498.9837,449.10715,431.09641,413.08624,369.06021,345.06033,315.04965,303.04959,285.03809,147.06531,85.02893,71.04981 | flavonoids -(1-2)-L-rhamnoside ^b^ | flavonoid glycosides |
| **47** | 13.54 | 595.16412/+ | C_27_H_30_O_15_ | 12.5 | -2.733 | 491.12286,433.11295,397.09204,353.06506,329.06549,299.05508,287.05478,129.05473,97.02885,85.02896,71.04984 | kaempferol-3-*O*-rutinoside ^b^ | flavonoid glycosides |
| **48** | 13.87 | 625.17487/+ | C_28_H_32_O_16_ | 12.5 | -2.305 | 479.11835,463.12247,360.07504,317.06540,302.04193,285.03906,85.02895,85.02895 | narcissoside ^b^ | flavonoid glycosides |
| **49** | 14.08 | 447.09286/- | C_21_H_20_O_11_ | 12.5 | 1.504 | 284.03217,255.02940,227.03410,179.05505,151.00217,101.02258,71.01198 | luteoloside ^*^ | flavonoids |
| **50** | 15.06 | 287.05502/- | C_15_H_12_O_6_ | 10.5 | 0.005 | 259.0603,243.06537,215.07063,201.05363,178.99692,174.95496,158.97711,146.95912,130.98178,125.02287,121.02761,95.01189,83.01203 | dihydrokaempferol ^a^ | coumarins |

Continued from **Table S2**

| **Comp.** | **RT (min)** | ***m/z***  **(pos/neg)** | **Formula** | **RDB** | ***δ* ppm** | **MS/MS**  **(*m/z*)** | **Identification** | **Category** |
| --- | --- | --- | --- | --- | --- | --- | --- | --- |
| **51** | 15.31 | 449.10739/+ | C_21_H_20_O_11_ | 11.5 | -0.997 | 313.82501,287.05469,111.04424,133.08588,153.01814,153.01814,268.71881,251.16347 | quercitrin ^*^ | flavonoids |
| **52** | 15.88 | 757.19678/+ | C_36_H_36_O_18_ | 18.5 | -0.872 | 653.14746,595.14496,449.10773,419.13379,345.06015,303.04953,257.08093,213.09169,165.05450,147.04398,119.04933,91.05468 | quercetin 3-*O*-*β*-D-(6''-*p*-coumaroyl) glucopyranosyl (1-2)-*α*-L-rhamnopyranoside ^b^ | flavonoid glycosides |
| **53** | 17.14 | 253.04973/- | C_15_H_10_O_4_ | 11.5 | 0.195 | 238.93736,217.84906,208.05136,186.85490,164.10629,149.02203,149.02203,135.00694,120.06226,85.94765 | 4',7-dihydroxyisoflavone ^a^ | flavonoids |
| **54** | 17.15 | 741.19995/+ | C_36_H_36_O_17_ | 18.5 | -3.475 | 433.11258,481.27368,397.09042,419.13342,329.06570,287.05457,257.08075,213.09041,165.05452,147.04398,119.04931,105.84399 | kaempferol 3-*O-β*-D-(6''-p-coumaroyl) glucopyranosyl (1-2)-*α*-L-rhamnopyranoside ^b^ | flavonoid glycosides |

Continued from **Table S2**

| **Comp.** | **RT (min)** | ***m/z***  **(pos/neg)** | **Formula** | **RDB** | ***δ* ppm** | **MS/MS**  **(*m/z*)** | **Identification** | **Category** |
| --- | --- | --- | --- | --- | --- | --- | --- | --- |
| **55** | 17.27 | 423.12918/- | C_20_H_24_O_10_ | 9.5 | 0.088 | 379.13840,349.09207,305.10342,261.11270,233.11761,209.04449,177.05460,147.04384,121.02805,81.03278,73.02799 | ginkgolide B ^*^ | ginkgolide |
| **56** | 17.34 | 407.13391/- | C_20_H_24_O_9_ | 9.5 | 0.251 | 379.13852,363.14426,351.14450,335.14972,319.15482,291.16028,273.14911,247.16977,229.15897,161.05933,117.06959, | ginkgolide A ^*^ | ginkgolide |
| **57** | 18.33 | 301.03479/- | C_15_H_10_O_7_ | 11.5 | 0.511 | 273.03931,245.04533,229.04936,193.01363,178.99736,169.01360,151.00232,121.02802,107.01246,83.01233,65.00191 | quercetin ^*^ | flavonoids |
| **58** | 20.40 | 245.11740/- | C_15_H_18_O_3_ | 7.5 | 0.179 | 231.14093,217.12294,197.90256,178.94695,161.05934,133.06464,110.29819,92.99979,91.02013,74.98893 | *α*-santonin ^a^ | ketolides |
| **59** | 20.47 | 271.06049/- | C_15_H_12_O_5_ | 10.5 | 1.439 | 177.01779,151.00252,119.04865,107.01205,96.66548,84.76134,65.00153 | naringenin ^*^ | flavanones |
| **60** | 20.79 | 269.04517/- | C_15_H_10_O_5_ | 11.5 | 2.677 | 227.03336,151.00244,117.03316,83.01223 | apigenin ^*^ | flavonoids |
| **61** | 21.26 | 285.03992/- | C_15_H_10_O_6_ | 11.5 | 0.556 | 229.04880,213.05502,185.05927,169.06543,154.89703,107.01220,83.01218,56.96959 | kaempferol ^*^ | flavonoids |

Continued from **Table S2**

| **Comp.** | **RT (min)** | ***m/z***  **(pos/neg)** | **Formula** | **RDB** | ***δ* ppm** | **MS/MS**  **(*m/z*)** | **Identification** | **Category** |
| --- | --- | --- | --- | --- | --- | --- | --- | --- |
| **62** | 21.78 | 315.05057/- | C_16_H_12_O_7_ | 11.5 | 0.641 | 300.02707,283.02536,271.02359,243.02847,180.89844,164.01012,151.00223,126.87975,83.01201,61.98664 | isorhamnetin ^*^ | flavonoids |

Notes: “*” indicates the comparison with the standard product, “a” indicates the comparison with the metlin database, “b” indicates the comparison with the Pub Chem, HMDB database

**Table S3** Standard curve regression equation of nine compounds

| Comp. | Regression equation | r^2^ | Linear range (ng·mL^-1^) | LOD  (ng·mL^-1^) | LOQ  (ng·mL^-1^) |
| --- | --- | --- | --- | --- | --- |
| 3,4-dihydroxybenzoic acid | Y=40.39614X-1.53599 | 0.9993 | 8-1000 | 4.00 | 6.00 |
| *p*-hydroxybenzoic acid | Y=0.489954X-0.90707 | 0.9994 | 2-500 | 0.29 | 1.40 |
| rutin | Y=0.489954X-0.143839 | 0.9995 | 16-4200 | 3.60 | 5.30 |
| astragalin | Y=0.243891X-0.786317 | 0.9990 | 2-500 | 0.10 | 1.50 |
| guanosine | Y=28.0581X-112.128 | 0.9997 | 4-1000 | 2.30 | 3.70 |
| uridine | Y=1.18502X-23.2492 | 0.9990 | 20-5000 | 8.00 | 16.00 |
| ginkgolide A | Y=0.0811572X-0.266992 | 0.9993 | 40-5000 | 3.20 | 6.40 |
| ginkgolide C | Y=0.0222128X-0.163991 | 0.9997 | 16-1600 | 1.30 | 8.00 |
| ginkgolide J | Y=0.181545X-0.0387335 | 0.9991 | 4-1000 | 2.00 | 4.00 |

**Table S4** Precision, repeatability and stability of nine compounds (*n* = 6)

| Comp. | Precision | | | Repeatability  (RSD, %) | Stability  (RSD, %) |
| --- | --- | --- | --- | --- | --- |
|  | Intra-day (RSD, %) | Intra-day (RSD, %) | |  |  |
| 3,4-dihydroxybenzoic acid | 1.96 | | 3.29 | 1.88 | 2.97 |
| *p*-hydroxybenzoic acid | 1.98 | | 1.82 | 2.91 | 1.51 |
| rutin | 2.79 | | 2.34 | 1.95 | 1.97 |
| astragalin | 3.48 | | 4.88 | 3.81 | 3.53 |
| guanosine | 1.76 | | 3.16 | 3.45 | 2.46 |
| uridine | 1.90 | | 3.02 | 3.05 | 3.09 |
| ginkgolide A | 1.31 | | 4.07 | 2.99 | 3.59 |
| ginkgolide C | 2.63 | | 4.53 | 2.56 | 1.47 |
| ginkgolide J | 2.22 | | 3.15 | 3.03 | 1.24 |

**Table S5** Sample recoveries and the RSD values of nine compounds

| Comp. | Add (ng) | Detection (ng) | Recovery (%) | RSD (%, *n* = 3) |
| --- | --- | --- | --- | --- |
| 3,4-dihydroxybenzoic acid | 284.18 | 640.71 | 100.45 | 1.61 |
|  | 355.00 | 715.46 | 101.47 | 1.30 |
|  | 426.27 | 778.90 | 99.39 | 2.12 |
| *p*-hydroxybenzoic acid | 72.72 | 163.04 | 99.21 | 3.03 |
|  | 90.90 | 183.34 | 101.69 | 4.26 |
|  | 109.08 | 195.88 | 96.24 | 3.94 |
| rutin | 1015.65 | 2232.30 | 94.78 | 0.39 |
|  | 1269.00 | 2573.38 | 102.74 | 0.30 |
|  | 1523.48 | 2799.31 | 100.41 | 0.94 |
| astragalin | 104.02 | 231.14 | 97.20 | 1.98 |
|  | 130.00 | 254.26 | 95.56 | 3.45 |
|  | 156.03 | 280.93 | 96.71 | 4.02 |
| guanosine | 326.96 | 726.15 | 97.09 | 0.75 |
|  | 409.00 | 804.00 | 96.65 | 2.86 |
|  | 490.44 | 884.34 | 96.94 | 1.07 |
| uridine | 612.00 | 1382.66 | 100.92 | 0.89 |
|  | 765.00 | 1568.00 | 104.96 | 2.37 |
|  | 918.00 | 1716.00 | 103.59 | 2.17 |
| ginkgolide A | 1066.40 | 2358.26 | 96.14 | 3.12 |
|  | 1333.00 | 2660.09 | 99.55 | 1.93 |
|  | 1599.60 | 2858.59 | 95.37 | 2.50 |
| ginkgolide C | 369.44 | 838.28 | 101.91 | 2.82 |
|  | 462.00 | 938.29 | 103.13 | 0.59 |
|  | 554.16 | 1041.27 | 104.56 | 3.14 |
| ginkgolide J | 203.44 | 462.80 | 102.48 | 2.84 |
|  | 254.00 | 517.16 | 103.49 | 2.75 |
|  | 305.16 | 567.13 | 102.51 | 4.12 |

**Table S6** Quantification of nine compounds in 13 batches of GAO

| batch number | Average concentration ± SD（ng·mL^-1^） | | | | | | | | |
| --- | --- | --- | --- | --- | --- | --- | --- | --- | --- |
|  | 3，4-dihydroxybenzoic acid | *p*-hydroxybenzoic acid | Rutin | Astragalin | Guanosine | Uridine | Ginkgolide A | Ginkgolide C | Ginkgolide J |
| 200603 | 23233.00±1714.08 | 12800.00±990.60 | 57645.50±1774.90 | 6951.00±497.849 | 12759.00±1097.79 | 41901.67±1550.94 | 54609.33±1573.16 | 26326.67±2206.10 | 12087.50±1601.69 |
| 201219 | 16780.33±315.61 | 9186.66±604.841 | 61350.00±4061.09 | 8177.00±447.26 | 14115.67±64.82 | 33111.67±15318.74 | 71320.83±3482.66 | 30045.00±1653.59 | 12003.33±416.72 |
| 201207 | 16632.00±622.78 | 9006.66±423.35 | 65640.00±1178.06 | 7073.33±1107.29 | 16780.33±315.61 | 33552.67±2100.87 | 69270.00±2106.25 | 30906.67±1819.13 | 12896.67±255.94 |
| 201228 | 17851.00±784.34 | 9610.00±249.79 | 62735.00±410.82 | 7266.66±292.24 | 15012.17±601.52 | 38566.67±797.37 | 69468.33±1152.14 | 29361.67±6282.80 | 13390.00±401.46 |
| 201218 | 16988.33±457.55 | 9106.66±531.53 | 65153.33±2990.87 | 7143.33±789.11 | 15711.67±263.92 | 37903.33±4034.75 | 68673.33±2081.35 | 32083.33±1751.88 | 13206.67±170.97 |
| 201208 | 15463.33±1072.76 | 10126.67±241.93 | 63773.33±2472.77 | 7035.00±1670.71 | 11000.00±198.68 | 29166.67±3062.46 | 66765.00±3167.59 | 29310.00±364.58 | 12291.67±428.90 |
| 201217 | 16443.33±448.11 | 8766.66±719.88 | 63006.67±3815.57 | 7638.33±581.06 | 20400.00±1018.68 | 39496.67±5528.80 | 67270.00±3812.48 | 27588.33±2356.58 | 12778.33±790.89 |
| 201226 | 17360.33±282.82 | 9376.66±220.52 | 64445.00±1904.19 | 7091.66±947.15 | 16978.33±405.16 | 36268.33±4543.84 | 70407.67±1022.33 | 31978.33±1332.05 | 13011.67±652.48 |
| 201222 | 17761.67±1512.61 | 9090.00±616.52 | 63478.33±1375.88 | 6501.66±742.97 | 20435.00±671.17 | 38250.00±4918.69 | 67603.33±2562.83 | 23090.00±12531.77 | 12715.00±766.82 |
| 201224 | 16501.67±922.58 | 9196.66±1143.08 | 63335.00±2260.21 | 7123.33±947.89 | 13143.33±533.13 | 23560.00±4711.39 | 69831.67±2665.00 | 30506.67±1007.87 | 12761.67±253.83 |
| 201212 | 18042.00±371.62 | 7940.00±821.64 | 60320.17±2749.91 | 7206.66±136.04 | 13760.00±186.81 | 30366.67±3004.06 | 67011.67±3286.55 | 27241.67±6519.40 | 12106.67±594.48 |
| 201205 | 16753.33±477.18 | 9046.66±766.31 | 56809.83±1252.95 | 7205.16±981.96 | 14361.67±177.78 | 34732.33±3856.90 | 70664.33±1479.96 | 27321.67±914.22 | 11675.00±278.79 |
| 201149 | 17101.67±687.68 | 10773.33±711.78 | 65018.33±3345.74 | 8383.33±698.21 | 10706.67±346.27 | 26485.00±3933.61 | 72193.33±3992.68 | 27183.33±5586.85 | 13213.33±727.08 |

**Table S7** Standard curve regression equation for five compounds

| Comp. | Regression equation | r^2^ | Linear range (μg·mL^-1^) | LOD (ng·mL^-1^) | LOQ  (ng·mL^-1^) |
| --- | --- | --- | --- | --- | --- |
| estradiol | Y=2.4786X-0.90707 | 0.9994 | 0.065-2 | 10.0 | 20.0 |
| chenodeoxycholic acid | Y=0.00341523X+0.19741 | 0.9947 | 0.131-2 | 1.0 | 2.0 |
| 2,6-diisopropylphenol | Y=0.000189033X-0.00243929 | 0.9990 | 0.064-1.95 | 5.0 | 10.0 |
| zidovudine | Y=0.0338931X-0.0552432 | 0.9997 | 0.016-2 | 0.1 | 1.0 |
| PG | Y=1.90871X-22.8361 | 0.9966 | 0.019-10 | 0.1 | 1.0 |

**Table S8** Precision and repeatability for five compounds (*n* = 3)

| Comp. | Precision (RSD, %) | Repeatability (RSD, %) |
| --- | --- | --- |
| estradiol | 1.66 | 3.29 |
| chenodeoxycholic acid | 2.29 | 4.90 |
| 2,6-diisopropylphenol | 3.55 | 4.88 |
| zidovudine | 2.02 | 4.78 |
| PG | 1.76 | 4.87 |

**Table S9** Identification the metabolites of PG in plasma

| RT  (min) | molecular formula | identification | MS/MS (*m/z*) |
| --- | --- | --- | --- |
| 8.60 | C_20_H_22_O_9_  (**M0**) |  | 405.11838, 243.06572, |
| 6.14  6.75 | C_26_H_30_O_15_  (**M1**) | piceatannol-3'-*O*-*β*-D-glucuronide | 581.15051,405.11880,243.06580,175.02388,113.02303, 85.02806;  581.15033, 419.09869, 243.06566, 175.02306, 113.02303, 85.02802 |
| 7.53 | C_20_H_22_O_12_S  (**M2**) | piceatannol-3'-*O*-*β*-D- sulfate | 485.07553, 323.02240, 243.06573, 175.07500; |
| 10.15 | C_21_H_22_O_10_  (**M3**) | piceatannol methylation- glucuronide | 433.11453，398.92725, 257.08145, 113.02322, 85.02771 |

**Table S10** Identification the metabolites of PG in bile

| RT (min) | molecular formula | identification | MS/MS (*m/z*) |
| --- | --- | --- | --- |
| 6.10  6.70 | C_26_H_30_O_15_  (**M1**) | piceatannol-3'-*O*-*β*-D-glucuronide | 581.15051, 405.11853, 243.06578, 113.02300, 59.01247;  581.15045, 419.09735, 405.12027, 243.06558, 113.02299, 59.01245;  581.15033, 419.09750, 405.12045, 243.06551, 113.02296, 59.01246 |
| 7.35 | C_20_H_22_O_12_S  (**M2**) | piceatannol-3'-*O*-*β*-D- sulfate | 485.07550, 405.12088, 323.02255, 243.06566, 79.95597;  485.07547, 405.11905, 323.02289, 243.06583, 201.05479, 113.02307. |
| 5.50  7.38 | C_26_H_30_O_18_S  (**M4**) | piceatannol-3'-*O*-*β*-D-glucuronide- sulfate | 661.10730, 499.05438, 485.07550, 323.02237, 243.06558, 113.02285, 79.95588;  661.10724, 499.05496,485.07520, 419.09875, 323.02280, 243.06563, 113.02302, 85.02807. |

**Table S11** Accuracy and precision of PG (*n* = 6)

| Comp. | Con. (μg/mL) | Intra-day | | Inter-Day | |
| --- | --- | --- | --- | --- | --- |
|  |  | Accuracy (RE, %) | Precision (RSD, %) | Accuracy (RE, %) | Precision (RSD, %) |
| PG | 0.005 | 85.50 | 8.93 | 118.44 | 10.83 |
|  | 5 | 118.21 | 2.35 | 117.72 | 7.28 |
|  | 30 | 105.11 | 8.77 | 99.82 | 12.54 |

**Table S12** Extraction recovery and matrix effect of PG (*n* = 6)

| Comp. | Con. (μg/mL) | Extraction recovery | | Matrix effect | |
| --- | --- | --- | --- | --- | --- |
|  |  | Mean ± SD (%) | RSD (%) | Mean ± SD (%) | RSD (%) |
| PG | 0.005 | 100.75±13.12 | 13.12 | 91.51±11.72 | 12.81 |
|  | 5 | 90.89±2.92 | 3.21 | 103.66±6.75 | 6.51 |
|  | 30 | 98.88±12.88 | 13.02 | 102.52±9.48 | 9.25 |

**Table S13** Stability of PG in rat blank plasma (*n* = 6)

| Comp. | Con.(μg/mL) | 4h | | 24h | | Freeze-thaws stability (3 times) | | | 15 days | |
| --- | --- | --- | --- | --- | --- | --- | --- | --- | --- | --- |
|  |  | Measured con. | RSD (%) | Measured con. | RSD (%) | | Measured con. | RSD (%) | Measured con. | RSD (%) |
| PG | 0.005 | 111.33 | 11.78 | 116.33 | 8.45 | | 107.66 | 8.66 | 92.00 | 11.92 |
|  | 5 | 117.12 | 7.48 | 115.03 | 9.61 | | 104.58 | 12.80 | 100.78 | 9.37 |
|  | 30 | 98.21 | 12.83 | 98.37 | 12.67 | | 105.11 | 8.77 | 96.48 | 7.35 |
